# Characterizing the trophic ecology of herbivorous coral reef fishes using stable isotope and fatty acid biomarkers

**DOI:** 10.1371/journal.pone.0327594

**Published:** 2025-06-30

**Authors:** Rita García-Seoane, W. Lindsey White, Brett M. Taylor, Kendall D. Clements

**Affiliations:** 1 Instituto Español de Oceanografía (IEO-CSIC), Centro Oceanográfico de A Coruña, A Coruña, Spain; 2 Department of Earth Sciences, University of Hawai‘i at Mānoa, Honolulu, Hawaii, United States of America; 3 Faculty of Health and Environmental Sciences, Auckland University of Technology, Auckland, New Zealand; 4 University of Guam Sea Grant & Marine Laboratory, Mangilao, GuamUnited States of America; 5 School of Biological Sciences, University of Auckland, Auckland, New Zealand; Ocean Frontier Institute, CANADA

## Abstract

Understanding the trophic ecology of herbivorous and detritivorous fishes is essential for evaluating their ecological roles in coral reef ecosystems. In this study, we combined bulk stable isotope (δ^15^N and δ^13^C) and fatty acid analyses to investigate trophic partitioning and dietary resource use among herbivorous and detritivorous fishes from the Great Barrier Reef, Australia. Isotopic niches and fatty acid profiles confirmed significant trophic partitioning among algivores, detritivorous surgeonfishes, and parrotfishes. We also applied mixing models based on these ecological tracers to quantify the contributions of basal dietary sources to the fish. Our findings further support previous dietary knowledge for several species, including algivorous acanthurids, kyphosid chubs, and the rabbitfish *Siganus doliatus*. However, they also highlight trophic niche specializations within these groups, particularly in *Naso unicornis*, which assimilates substantial dietary protein from epiphytic cyanobacteria despite a macroalgal diet, and in the detritivorous *Ctenochaetus striatus*, which exhibited isotopic similarities to parrotfishes but differed in fatty acid composition, likely due to a higher intake of diatoms. Additionally, our analyses reinforce the distinctive dietary composition of parrotfishes, emphasizing the complexity of their feeding biology, in which microscopic photoautotrophs such as cyanobacteria and dinoflagellates play a key dietary role that has often been overlooked in previous studies on their nutritional ecology. Furthermore, these findings underscore the usefulness of multi-tracer approaches in refining our understanding of coral reef fish trophic ecology.

## Introduction

The study of herbivory has proceeded along distinct trajectories in different ecological contexts. In most terrestrial and freshwater systems, herbivory has been studied from an animal-based perspective [1], where it is defined and measured in terms of the contribution of plant biomass to the nutritional requirements of animals. In contrast, in coral reef systems, herbivory has been largely studied from a plant-based perspective, focusing on the consumption or removal of plant material, irrespective of its nutritional contribution to the consumer [2–4]. As a result, our understanding of herbivory on coral reefs, and trophodynamics more generally, may be incomplete, especially given the complexity of available resources [5,6].

Herbivorous fishes are the most conspicuous and well-studied herbivores on coral reefs; they are abundant and generally considered to be major agents in structuring benthic communities [7–9]. Traditional methods, such as gut content analysis, can provide valuable insights into the diet of some herbivorous reef fish species [10,11]. However, many species have a trituration mechanism, such as a pharyngeal mill or a gizzard-like stomach, that makes identification and quantification of gut contents problematic [12]. More recently, histological analysis of bite cores [13,14] and molecular DNA metabarcoding of ingesta [9,15] and bite cores [16,17] have further advanced our understanding of diet in some species, and emphasized the importance of using multiple methodological approaches.

However, significant knowledge gaps remain regarding resource partitioning among grazing taxa, especially parrotfishes and surgeonfishes, that appear to target microscopic photoautotrophs or detrital biomass [12,13,16,18]. Additionally, some parrotfish species traditionally classified as herbivorous may actually target sessile invertebrates such as sponges and ascidians, perhaps because they contain microbial biomass [12,17]. Are most coral reef grazing taxa generalist feeders on turf assemblages [19,20], or is resource partitioning fine-grained, similar to patterns observed in freshwater grazing fish faunas [21,22]? Addressing these questions poses challenges due to the limitations of conventional approaches and the inherent complexity of coral reef food webs, which are characterized by phylogenetically diverse and complex mixtures of macroscopic and microscopic primary resources [5,12,23].

The use of stable isotopes (SI) of carbon (δ^13^C) and nitrogen (δ^15^N) in bulk tissues offers a valuable method for tracing primary production sources and resolving resource partitioning through isotope niche analysis and mixing models [24,25]. This approach can be enhanced by incorporating fatty acid (FA) biomarkers [26–28], which can differentiate the contributions of various production sources, including diatoms, dinoflagellates, bacteria, and animal-derived material, to fish diets [29]. The FA composition can also be used in mixing models to estimate food source contributions to consumers [30]. Therefore, integrating FA and SI analyses presents a robust and comprehensive framework for assessing resource partitioning and nutritional inputs in consumers with complex feeding behaviors and diets, such as grazing coral reef fishes.

An approach that combines FA with SI analyses also allows for differentiation between what is ingested and what is actually assimilated, and thus of nutritional value to fish consumers. Biomarker approaches have been widely applied to determine the main nutritional sources for herbivorous freshwater fishes [26]. In freshwater ecosystems, microphytobenthos (i.e., benthic photoautotrophic cyanobacteria and eukaryotic microalgae) associated with sediment, epilithic, and epiphytic periphyton on aquatic angiosperms constitute important food resources for grazing fishes such as gizzard shad (*Dorosoma cepedianum*) [31], Altai Osman carp (*Oreoleuciscus potanini*) [32], and suckermouth armoured catfishes (Loricariidae) [33]. These studies emphasize the concept of “peanut butter and crackers feeding”, where ingested plant material may be of little nutritional value but is consumed because it serves as a substrate for nutrient-rich microbial elements, such as diatoms and cyanobacteria [31].

On coral reefs, microphotoautotrophs are abundant on substrata grazed by herbivorous fishes [13,16] and are also conspicuous in DNA metabarcoding sequences of ingesta from these fishes [9,15]. Biomarker evidence suggests that many coral reef herbivores assimilate microphotoautotrophs [9,12,34], yet only one study to date has attempted to integrate SI and FA analyses to examine the links between primary production and herbivorous fish on a coral reef [35]. Fey and colleagues investigated various consumer species in the Marquesas Islands, including two detritivorous/herbivorous surgeonfishes (*Acanthurus nigricans* and *Ctenochaetus marginatus*) and two parrotfishes (*Scarus koputea* and *S. rubroviolaceus*). They detected diatom FA markers in both surgeonfishes, a dinoflagellate FA marker in the two parrotfishes, and bacterial/cyanobacterial markers in all four grazing fish species [35]. However, they found no important contribution of phytoplankton-derived organic matter to the diets of these surgeonfishes and parrotfishes, suggesting that the presence of these biomarkers in the fish may have originated from dead phytoplankton accumulating on reef surfaces. The significance and prevalence of microphotoautotroph assimilation in grazing coral reef fishes thus remain open questions.

In this study, we seek to infer trophic partitioning and identify nutritional sources in a range of nominally herbivorous and detritivorous coral reef fishes from the Great Barrier Reef, Australia. We used an integrative approach that triangulates SI and FA analyses of fish tissues and potential food resources with available information from stomach content analysis on taxa lacking a mechanical trituration mechanism. This includes browsing species for which macroscopic food items can be reliably quantified [10,11]. The combination of SI and FA markers also allowed us to: a) identify taxa with higher trophic levels linked to omnivory, such as those consuming sessile filter-feeding invertebrates [12,36]; and b) discriminate among various types of microphotoautotrophs, including dinoflagellates, diatoms, and diazotrophic cyanobacteria [37–39].

## Materials and methods

### Sample collection

**Fig 1:**
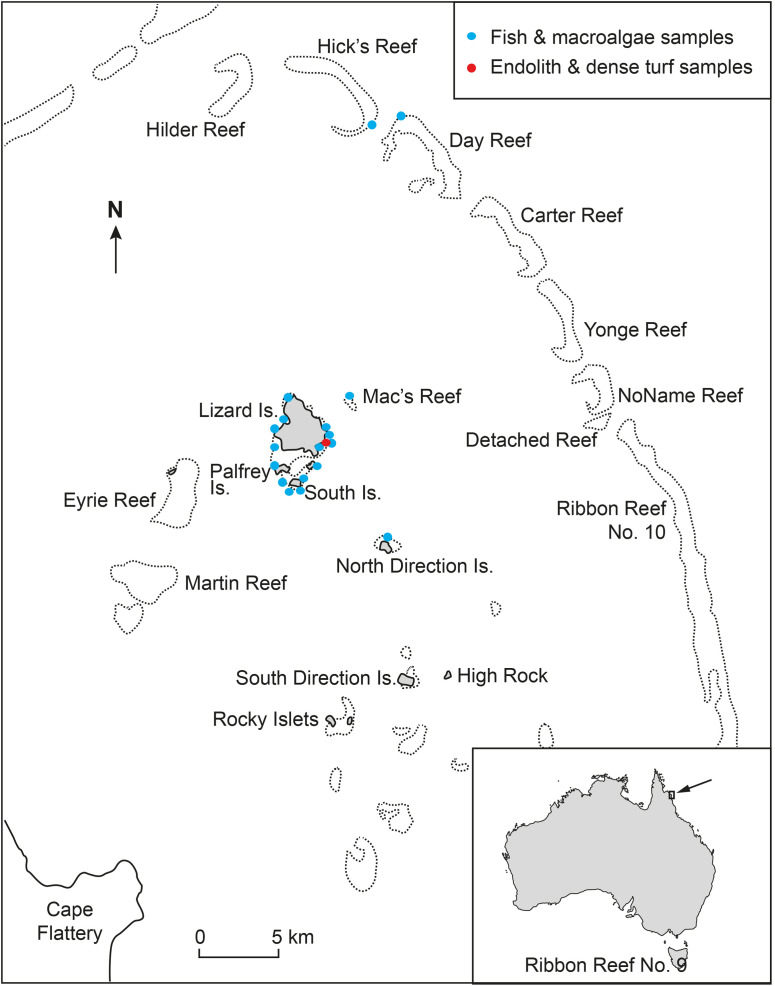
Map showing sampling locations of fish and organic matter sources at the Lizard Island Complex and adjacent outer reefs, Great Barrier Reef, Australia.

Individuals from 18 species of nominally herbivorous coral reef fishes were collected in December 2014 (Austral summer) at mid-shelf sites of the Lizard Island Complex and adjacent outer reef sites, Great Barrier Reef, Australia (14º40’4.13”S, 145º27’45.14”E) (Fig 1). Sampling sites were selected based on the known distribution and abundance of species of interest, especially parrotfishes [10,40]. Fishes (n = 111 specimens, see S1 Table) were collected by spearing on snorkel. Acanthurid (Acanthuridae) taxa investigated were the surgeonfishes *Acanthurus lineatus*, *A. nigrofuscus*, *Ctenochaetus striatus* and *Zebrasoma velifer*, and the unicornfishes *Naso tonganus* and *N. unicornis*. Parrotfishes (Labridae-Scarinae) were represented by six scraping (*Scarus frenatus*, *S. ghobban*, *S. niger*, *S. rivulatus*, *S. spinus*, and *S. schlegeli*) and two excavating species (*Chlorurus microrhinos* and *C. spilurus*). Kyphosid chubs (Kyphosidae) were represented by *Kyphosus cinerascens* and *K. vaigiensis*, angelfishes (Pomacanthidae) by *Pomacanthus sexstriatus*, and rabbitfishes (Siganidae) by *Siganus doliatus*. All fish specimens collected for this study were sexually mature adults with the exception of some individuals of *N. tonganus*, *N. unicornis*, *Z. velifer*, and *C. microrhinos*. These subadult individuals are close to size at sexual maturity [41–43], and so all samples used in this study would reflect the adult diet of each fish species.

The potential major sources of organic matter for these fish were also sampled (n = 92 samples, see S2 Table) at the same sites when present (Fig 1). These sources comprised five end-member categories: dense turf, endoliths and dense turf, endoliths and sparse turf, coral rubble containing endoliths, and macroalgal species belonging to Phaeophyceae: *Padina boergesenii* and *Turbinaria conoides*, Chlorophyta: *Dictyosphaeria versluysii* and *Halimeda macrophysa*, and Rhodophyta: *Galaxaura marginata*, *Ganonema farinosum*, *Halymenia durvillei*, *Ohelopapa flexilis*, *Polysiphonia* sp., *Portieria hornemannii*, and *Hypnea valentiae*. Dense turf, often termed epilithic turf in the literature, is a complex assemblage of filamentous algae, cyanobacteria, diatoms, detritus, and potentially other sources, serving as a primary source of organic matter on coral reefs [23,44,45]. This source is typically considered a homogeneous end-member category, distinct from macroalgae in studies of herbivorous reef fish trophic ecology [7,46]. The source categories endoliths and dense turf and endoliths and sparse turf consisted of a mixture of epilithic turf and the underlying euendolithic biota [12,47]. Both were collected using a hammer and chisel (S1 Fig). Coral rubble containing endoliths referred to bioeroded fragments of *Acropora* sp. staghorn coral, 10–15 mm in diameter, that were collected from the reef flat substratum. Subtropical and tropical brown macroalgae often have high epiphyte loads [48–50]. Since browsing fishes are expected to ingest associated epiphytes with macroalgal thallus, macroalgae were not cleared of epiphytes before analysis, ensuring that the FA composition reflected the combined food resource. In the case of the brown alga *T. conoides*, we analyzed the thallus and reproductive tissue separately, as some browsing fishes specifically target the reproductive tissues of brown algae [51,52].

Sample collection was permitted by the Great Barrier Reef Marine Park Authority (permit nº: G14/37216.1) and the Queensland Department of Agriculture, Fisheries and Forestry fisheries (permit nº: 170251). Sampling was performed in compliance with James Cook University guidelines and regulations for ethical treatment of animals (approval nº: A2027). No coral habitat was degraded during this research.

### Tissue sampling and preparation

Fish were killed by pithing immediately after capture, and transported on ice, along with source samples, to the laboratory at the Lizard Island Research Station (LIRS). Samples of white muscle tissue (~ 20 g wet weight) were taken from the dorsal pterygiophores. Both muscle and organic matter source samples were frozen in liquid nitrogen and subsequently stored at −20°C. The samples were freeze-dried and stored at room temperature until further analyses. For each individual, standard length (mm), fork length (mm), and total weight (g, wet weight) were recorded. Sex was determined through visual inspection of the gonads or, in the case of parrotfishes, by assigning color phases. S1 Table provides details of the observed ranges for each fish descriptor.

Upon arrival at the School of Biological Sciences, University of Auckland, samples were cryogenically ground using a MM301 Mixer Mill (Retsch). Inorganic carbon in the form of carbonates (CaCO_3_) can interfere with isotope (δ^13^C and δ^15^N) measurements unless removed from the sample before analysis [53,54]. To eliminate carbonates, samples with potentially high CaCO_3_ concentrations (i.e., dense turf, all endolith samples, and the macroalgae *G. marginata*, *G. farinosum*, *H. macrophysa*, and *P. boergesenii*) were treated with ~25 mL of 10% HCl until no more CO_2_ was released [55]. The treated samples were then dried at 60ºC before stable isotope (SI) analysis. To assess potential isotopic effects of acidification, five lipid-extracted fish samples (see below) with known C and N isotopic compositions were processed in the same manner. A non-parametric Wilcoxon signed-rank test revealed no significant differences between acid-treated and untreated samples for either δ^13^C (*V* = 15, *p* = 0.062) or δ^15^N (*V* = 10, *p* = 0.625) values. Therefore, all SI measurements were conducted on the acidified samples.

### Inclusivity in global research

Additional information regarding the ethical, cultural, and scientific considerations specific to inclusivity in global research is included in the Supporting Information (S1 Checklist).

### Bulk stable isotope analysis

Subsamples (~3 mg dry weight) of fish tissue and the organic matter sources were packed into tin capsules for bulk C and N isotope analysis. Bulk δ^13^C and δ^15^N values, along with mass percent C and N, were measured using a PDZ Europa ANCA-GSL elemental analyzer interfaced with a PDZ Europa 20–20 isotope ratio mass spectrometer (Sercon Ltd., Cheshire, UK) at UC Davis Stable Isotope Facility. Isotopic abundances were expressed in δ notation (‰) relative to VPDB (Vienna PeeDee Belemnite carbonate) for C and atmospheric N_2_ for N. In-house reference materials (USGS41 L-glutamic acid, Glutamic Acid, Bovine Liver, and Nylon) were analyzed in each analytical batch [56]. These reference materials were previously calibrated against international certified standards, including IAEA-600, USGS-40, USGS-41, USGS-42, USGS-43, USGS-61, USGS-64, and USGS-65. The precision (± se) of replicate within-run isotopic determinations of standards (n = 131) was < 0.03‰ for δ^13^C and <0.05‰ for δ^15^N. The analytical offset between reference and measured values was < 0.04‰.

Since lipids are depleted in ^13^C by 6–8‰ relative to proteins [57,58], all fish muscle and source samples were lipid-extracted using a 1:1 v/v mixture of trichloromethane:methanol [59] before δ^13^C analysis. In contrast, the δ^15^N analysis was performed on untreated aliquots, as lipid removal may alter N isotope values [58].

### Fatty acid analysis

The fatty acid (FA) composition of lipids was analyzed on aliquots of fish muscle tissue and a selected set of organic matter source samples (n = 44) (see S1 Appendix) at the Mass Spectrometry Centre, School of Biological Sciences, University of Auckland. Direct transesterification of dry tissues was performed following a modified version of the method described by Lepage and Roy [60]. Full details of sample preparation for FA analysis are provided in S1 Appendix. Individual fatty acid methyl esters (FAME) were identified and semi-quantified by gas chromatography-mass spectrometry (Agilent 7890B GC, 5977A MS) equipped with a Fused Silica Rtx®-2330 capillary column (100 m x 0.25 mm ID, 0.2 µm film, Shimadzu). The instrument parameters were set according to Smart and colleagues [61] (see S1 Appendix for details). Identification of FAME was based on retention times and mass spectra of chromatographic peaks, compared against an in-house library of 52 FAME using the software AMDIS (http://www.amdis.net/) [61]. Background FAME, typically excluded by AMDIS, were identified using the R package *MassOmics* [62]. Those FAME not included in this library were manually identified using the NIST 2014 Mass Spectral Library. Fatty acids were reported using standard shorthand nomenclature (C:Dn-X), where C represents the number of carbon atoms, D denotes the number of double bonds, and X indicates the position of the first double bond from the methyl terminal end. Data were normalized to internal standards and further normalized by biomass (g). Fatty acid composition was expressed as proportional data, representing the percentages of the total FA mass quantified in each sample.

Fatty acids were classified according to their chemical structure into saturated (SFA; e.g., 16:0), monounsaturated (MUFA; e.g., 16:1n-7), polyunsaturated (PUFA; e.g., 18:3n-3), and branched FA (BrFA; e.g., iso-15:0). PUFA with a terminal end ω-3 (n-3) or ω-6 (n-6) indicate essential FA, meaning they cannot be synthesized de novo by consumers and must be obtained from the diet [29]. In particular, essential long-chain (≥ C_20_) PUFA, i.e., 20:4n-6 (arachidonic acid, ARA), 20:5n-3 (eicosapentaenoic acid, EPA), and 22:6n-3 (docosahexaenoic acid, DHA), play a major role in the organism’s energy reserves as well as in physiological regulation and somatic growth [63]. We examined FA that were either the most abundant or exhibited the greatest variation across the organic matter sources and fish taxa studied. Our focus was on fatty acid trophic markers (FATM) of importance in trophic studies for interpreting variability in fish diets [29,38,63]. Specifically, we distinguished between FA indicative of diatoms (e.g., 14:0, 16:1n-7, EPA, and the ratios 16:1n-7/16:0, Σ16:0/Σ18:0, and DHA/EPA < 1), dinoflagellates (e.g., 18:1n-7, Σ18:0, DHA, and the ratio DHA/EPA > 1), bacteria/cyanobacteria (e.g., 17:0, 18:1n-7, BrFA, and the 18:1n-7/18:1n-9 ratio), and Phaeophyceae (e.g., 18:1n-9).

### Data analysis

#### Statistical tests.

Normality of bulk C and N isotopic values was tested using the Lilliefors-corrected Kolmogorov–Smirnov test, while homoscedasticity was assessed with Levene’s test. After confirming normality assumptions, a one-way ANOVA (General Linear Model with Type I error) was used to compare mean δ^13^C and δ^15^N values across organic matter sources and fishes. Pairwise differences were evaluated using Tukey’s HDS post-hoc test, with adjustments for unequal sample sizes. A one-way Multivariate Analysis of Variance (MANOVA), followed by multiple pairwise comparisons, was conducted to explore differences in the isotope composition of C and N across organic matter sources and fishes. The δ^13^C values of *D. versluyii* (see S2 Table) were outside the expected natural range for algae samples (δ^13^C > -11‰, [64]), indicating biochemical alteration of the C content in these samples. Consequently, data from *D. versluyii* were excluded from ANOVA, MANOVA, and post-hoc tests. The fish species *S. spinus* was also excluded from these tests due to the availability of only a single data point.

Since the FA data did not meet normality assumptions (performed the Lilliefors-corrected Kolmogorov–Smirnov and Levene’s tests), Kruskal-Wallis and Dunn’s post hoc tests were used to assess significant differences in FA composition among organic matter sources and fishes. *Scarus spinus* was also excluded from these tests. Variation in the FA profiles was further examined using linear discriminant function analysis (LDA) on Euclidean dissimilarity matrices derived from square-root transformed proportional data. Multivariate analysis was then applied to identify differences among groups outlined by the LDA, including a one-way PERMANOVA with multilevel pairwise comparisons. Similarity percentages analysis (SIMPER) was used to identify the FA contributing most to dissimilarities among significantly different groups (cut-off: 70%).

Finally, non-parametric Spearman’s rank correlations were computed to examine relationships between isotopic variables, between isotopic variables and fish morphometric variables, and between isotopic variables and FA. Statistical analysis was performed using PAST 4.03 [65], or R 4.1.1 [66] for PERMANOVA and multilevel pairwise tests, utilizing the “adonis()” and “pairwise.adonis()” functions from the package *vegan* [67,68]. All *p*-values were adjusted using the Bonferroni correction for multiple comparisons.

#### Mixing models.

We used Bayesian mixing models to evaluate the relative contribution of different organic matter sources to the fish diets. The models were implemented in R using the package *MixSIAR* [69]. We ran individual models for SI (δ^13^C and δ^15^N) [70], and for FA (the 42 FA detected in the samples) [30]. For each biotracer type, consumer data were input at the individual level (i.e., one record per organism). Source SI values were also input individually, whereas source FA values were entered as mean ± sd. The models were run using the Markov Chain Monte Carlo method with three parallel chains and 30^5^ iterations at the “normal” length setting. Model convergence was assessed using Gelman-Rubin and Geweke diagnostics. Since no prior information is available on the contribution of different sources to fish diets in this ecosystem, the models were run with uninformative priors (i.e., equal weight assigned to each source) and no random effects. The posterior distributions from the MixSIAR analyses were reported as the median and the 90% Bayesian credibility interval of diet proportions.

Mixing models require adjustments to account for the trophic shift in biotracer values from diet to consumer. These adjustments are commonly referred to as trophic discrimination factors (TDF) for SI [25] and calibration coefficients (CC) for FA [71]. For the SI models, we applied a TDF of 4.8 ± 1.2‰ (mean ± sd) for δ^15^N, based on estimates for herbivorous fish, including acanthurid species related to those studied here [72]. For δ^13^C, we used a TDF of 0.4 ± 1.3‰, a commonly adopted value across many taxa [73]. We specifically selected a δ^15^N TDF for herbivorous fish because it provided a better alignment between the isotope mixing space of the sources and our consumer data, compared to the more generalized literature TDF of 3.4 ± 1.0‰ [73], which is low for herbivorous fish (see Results section). However, no species-specific diet-tissue CC are available for the sampled fish, nor for other related marine species with similar dietary sources, either from natural conditions or laboratory feeding experiments. The limited literature on CC in marine fishes (see the recent review of [74]) reports values for taxa distantly related to those in our study. Given the conserved nature of CC within consumer taxa [26], applying non-specific CC would not be appropriate. Additionally, recent studies have shown that FA models without CC can yield robust diet estimations even without CC [28,75]. For these reasons, we chose not to use CC from the literature and instead set CC to zero in our FA models.

A major assumption of MixSIAR is that source pools must be statistically distinct [24,25]. To evaluate this, we assessed differences in biotracer values among sources both visually and statistically (see Results section for details). Visual inspection of organic matter sources in isotope space revealed significant overlap between macroalgae and coral rubble containing endoliths, and also between endoliths and dense turf and endoliths and sparse turf. The high degree of isotopic overlap among sources limits MixSIAR’s ability to accurately estimate their contributions to consumers. A MANOVA test further confirmed that these sources were not significantly different and could not be statistically distinguished. Besides, isotope models with two tracers can adequately differentiate a maximum of three sources. Beyond this, the models become undetermined [28]. To ensure model performance, we categorized basal sources into three groups: i) dense turf, ii) endolithic categories (pooling endoliths and dense turf and endoliths and sparse turf categories), and iii) macroalgae. Coral rubble containing endoliths was not included in the endolithic categories group because it overlapped with macroalgae in isotope space and was significantly different from the other two endolithic groups. Furthermore, the isotopic composition of *D. versluyii* differed notably from the macroalgae group, and therefore was excluded from the macroalgae source data input. We then verified that these three source groups adequately described our consumers isotopically using the Isotope Mixing Polygon Simulation developed by Smith and colleagues [76]. To visualize the FA composition of the sources and confirm that consumers are contained within the mixing space of the endmembers, we used LDA. This analysis showed that the three groups containing endoliths form a single cluster and could not be distinguished based on their FA profiles, a finding further supported by a non-significant PERMANOVA test. Accordingly, FA models were also run considering three sources: dense turf, the three pooled endolithic categories, and macroalgae. However, it is important to note that the specific endolithic sources included in the endolithic category differed between the isotope and FA models, meaning that direct comparisons of model outputs should be interpreted with caution.

## Results

### Isotopic analysis

**Fig 2:**
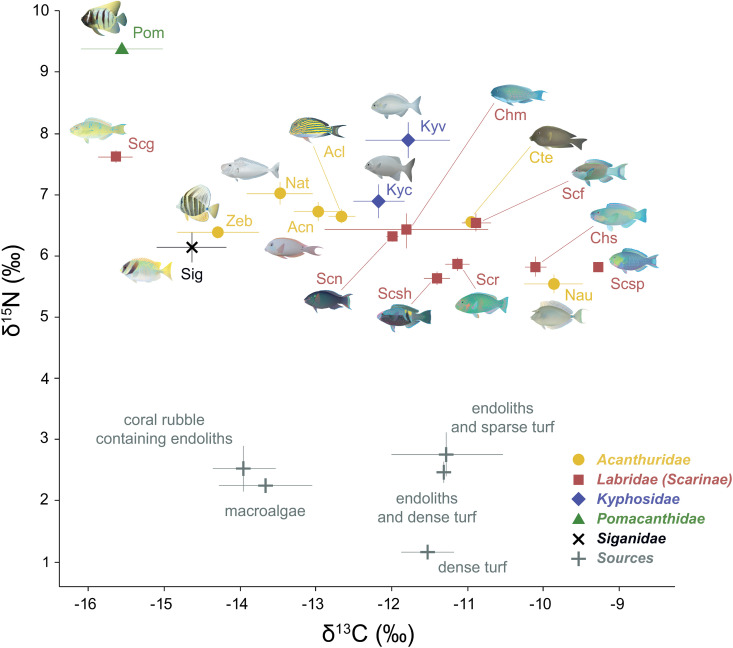
Bulk δ^15^N and δ^13^C values (‰, mean ± se) of eighteen fish species and the dominant organic matter sources in the food web collected near the Lizard Island Complex, Australia. The fish species belong the families Acanthuridae (Acl: *Acanthurus lineatus*, Acn: *A. nigrofuscus*, Cte: *Ctenochaetus striatus*, Nat: *Naso tonganus*, Nau: *N. unicornis*, Zeb: *Zebrasoma velifer*), Kyphosidae (Kyc: *Kyphosus cinerascens*, Kyv: *K. vaigiensis*), Pomacanthidae (Pom: *Pomacanthus sexstriatus*), Labridae-Scarinae (Chm: *Chlorurus microrhinos*, Chs: *C. spilurus*, Scf: *Scarus frenatus*, Scg: *S. ghobban*, Scn: *S. niger*, Scr: *S. rivulatus*, Scsh: *S. schlegeli*, Scsp: *S. spinus*), and Siganidae (Sig: *Siganus doliatus*). The sources analyzed were grouped into three clusters (dense turf, macroalgae + coral rubble containing endoliths, endoliths and dense turf + endoliths and sparse turf) according to their isotopic composition. Note that samples from *Dictyosphaeria versluysii* were excluded from this figure. Individual values and corresponding sample sizes are provided in S2 Table.

The δ^15^N values of consumers were 2.5 to 9 times higher than those of their baseline organic matter sources (Fig 2, S2 Table). The isotopic signals of the organic matter sources showed substantial overlap, although significant differences were observed between dense turf, macroalgae + coral rubble containing endoliths, and endoliths and dense turf + endoliths and sparse turf (MANOVA; Pillai’s trace = 0.59, F_4,164 _= 17.31, *p* < 0.001). Among sources, dense turf showed significantly lower δ^15^N values (~1.5‰ more negative) compared to other categories (ANOVA, F_4,80 _= 17.43, *p* < 0.001). However, no significant differences in δ^13^C values were found among sources due to high within-group variability, particularly in macroalgae (Fig 2, S2 Table), resulting in overlapping distributions. In the fish muscle, mean bulk δ^15^N ranged from 5.6‰ in *N. unicornis* to 9.4‰ in *P. sexstriatus*, while mean δ^13^C values ranged from −15.6‰ in *S. ghobban* to −9.6‰ in *S. spinus* (Fig 2, S2 Table). Although δ^13^C values exhibited greater intraspecific variability than δ^15^N, a significant negative correlation was observed between them (Spearman’s ρ = −0.52, *p* < 0.001). No significant correlations were found between δ^13^C values and fish fork length or weight (Spearman’s ρ = 0.16, *p* = 0.169; ρ = 0.15, *p* = 0.122, respectively), nor between δ^15^N and fork length (ρ = 0.12, *p* = 0.188). However, δ^15^N showed a weak but statistically significant positive correlation with fish weight (ρ = 0.19, *p* < 0.05).

Overall, δ^15^N values decreased across fish families in the following order: pomacanthids, kyphosid chubs, acanthurids, siganids, and parrotfishes, although significant differences were found only among *P. sexstriatus*, kyphosid chubs, and the other families (Tukey’s HDS after significant ANOVA, F_4,105 _= 30.42, *p* < 0.001). Differences in δ^15^N were also detected among species within the same family (Tukey’s HDS after significant ANOVA, F_16,92 _= 24.25, *p* < 0.001). For instance, *S. ghobban* showed significantly higher δ^15^N values than acanthurids and other parrotfish species, while *N. unicornis* had significantly lower δ^15^N values compared to other acanthurids. Although parrotfishes were generally more enriched in ^13^C than other groups, their δ^13^C values did not differ significantly from those of Acanthuridae or Kyphosidae. However, these three groups showed significant higher δ^13^C values than Pomacanthidae and Siganidae (Tukey’s HDS after significant ANOVA, F_4,105 _= 7.93, *p* < 0.001). Unlike the trend observed for δ^15^N, the δ^13^C values in *S. ghobban* were significantly lower than those observed in acanthurids and other parrotfishes, whereas *N. unicornis* had significantly higher δ^13^C values than other acanthurids (except *C. striatus*).

The δ^13^C and δ^15^N values indicated clear isotope niche segregation in certain species (MANOVA; Pillai’s trace = 1.45, F_32,184 _= 15.22, *p* < 0.001), particularly in *P. sexstriatus* and *S. ghobban*, while others, especially acanthurids and parrotfishes, showed high niche overlap (Fig 2, S2 Table). Similarly, isotope profiles differed significantly among fish families (MANOVA; Pillai’s trace = 0.78, F_8,210 _= 17.06, *p* < 0.001), except between acanthurids and parrotfishes (pairwise MANOVA, *p* = 3.127).

The C:N ratio in fish muscle was consistently around 3.1, whereas in the organic matter sources it ranged from 5 to 20, except for *T. conoides* samples, which were typically >25 (S2 Table).

**Fig 3:**
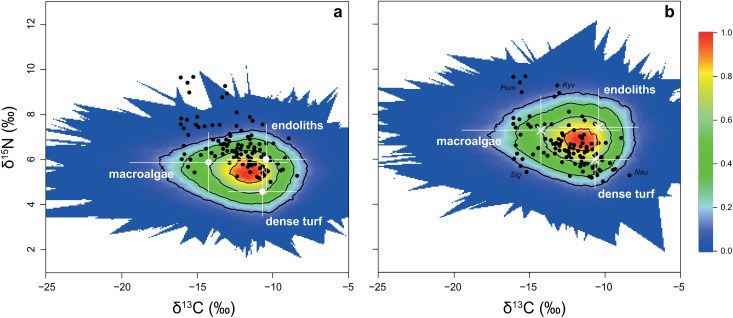
Stable isotope biplots depicting the results of Monte Carlo-simulated mixing source polygons for δ^13^C and δ^15^N values in reef fish (black dots) and mean isotopic ratios of potential sources (white crosses: dense turf, macroalgae, and endoliths = endoliths and dense turf + endoliths and sparse turf). Source values were corrected using either (a) generalized trophic discrimination factors (TDF) from Post [73] (0.4 ± 1.3‰ for δ^13^C and 3.4 ± 1.0‰ for δ^15^N), or (b) a combination of the δ^13^C TDF from Post [73] with a δ^15^N TDF of 4.8 ± 1.2‰ derived from Mill and colleagues [72]. Error bars represent 95% confidence intervals and incorporate the error in both source isotopic signatures and TDF. The 95% mixing region is delineated by the outermost contour (5% threshold), and the color scale indicates the probability that the proposed mixing model accurately estimates source contributions to consumers. In b, consumer samples falling outside the mixing region include *Pomacanthus sexstriatus* (Pom, n = 5), *Kyphosus vaigiensis* (Kyv, n = 2), *Siganus doliatus* (Sig, n = 2), and *Naso unicornis* (Nau, n = 1).

According to the simulated mixing polygons (Fig 3), 76% of the fish samples lay within the 95% mixing region when using a generalized δ^15^N TDF (Fig 3a), while this percentage increased to 91% when applying a herbivorous fish-specific δ^15^N TDF (Fig 3b). These simulations validated the use of these source clusters to explain consumer isotope signatures in MixSIAR and demonstrated that taxa-specific TDF (Fig 3b) provided a better correction for source values than generalized TDF (Fig 3a). However, although the assumption of the model was met in Fig 3b, some consumer data points fell outside the mixing region. This suggests that an alternative model (incorporating different sources, TDF, or other modifications) would be necessary to explain their isotopic signatures. To calculate a logical MixSIAR model, these data points were excluded from the dataset before analysis.

### Fatty acid analysis

A total of 42 FA were detected across all samples, with 21 exhibiting relative abundances greater than 1%, collectively accounting for 92–99% of the total FA in the dataset (see S3 and S4 Tables for detailed FA composition of sources and fishes, respectively). The dominant FA type in both organic matter sources and fishes was SFA, with 16:0 being the most represented (52 ± 4% in sources and 42 ± 1% in fishes), followed by MUFA and PUFA. The most abundant MUFA were 16:1n-7 in sources and 18:1n-9 in fishes, whereas LIN in sources and ARA in fishes contributed the most to the overall PUFA composition.

The LDA effectively separated dense turf, macroalgae, and the three endolithic sources pool in the space plot based on their FA profiles (S2 Fig). No significant differences were detected among endolithic categories (PERMANOVA, F_2,13 _= 0.65, *p *= 0.574), but significant differences were observed among dense turf, macroalgae, and endoliths (PERMANOVA, F_2,23 _= 5.75, *p* < 0.001). SIMPER analysis identified 12 FA (16:0, 14:0, 18:1n-9, ALA, 24:0, 16:1n-7, 18:0, ARA, 18:1n-7, LIN, 22:0, and iso-15:0, in this order) as the main contributors (>70%) to the observed differences in the LDA (37% overall average dissimilarity among groups) (see vectors in S2 Fig). For instance, dense turf showed significantly higher levels of 14:0 and 16:1n-7, while also exhibited the lowest percentage of 16:0 and the highest percentage of ALA (along with macroalgae) and EPA among all sources. Nevertheless, macroalgae reached the highest significant contributions of 18:1n-9, 22:0, and other PUFA but showed the lowest contributions of certain SFA (e.g., 18:0, iso-15:0, iso-16:0) and MUFA (e.g., 18:1n-7). The FA composition of endolithic categories varied, resembling either dense turf or macroalgae depending on the specific FA, though they exhibited the highest contributions of 18:1n-7 and BrFA. Significant differences were observed also among sources for other FATM, such as Σn-3/Σn-6 (highest in dense turf, lowest in coral rubble containing endoliths) and BrFA (highest in endoliths and dense turf and endoliths and sparse turf, lowest in macroalgae). However, no significant differences were observed in ΣSFA, ΣMUFA, or ΣPUFA among the five end-member sources (S3 Table). Significant differences in FA composition were also identified among macroalgal families (PERMANOVA, F_2,41 _= 10.66, *p* < 0.001), with brown macroalgae (Phaeophyceae) being distinguished from Chlorophyta and Rhodophyta (which were not significantly different in most cases). Phaeophyceae had higher proportions of 14:0, 15:0, 20:0, and most MUFA and PUFA (especially ARA), but lower proportions of 16:0, Σ16:0/Σ18:0, and BrFA (see S3 Table for pairwise comparisons).

**Fig 4:**
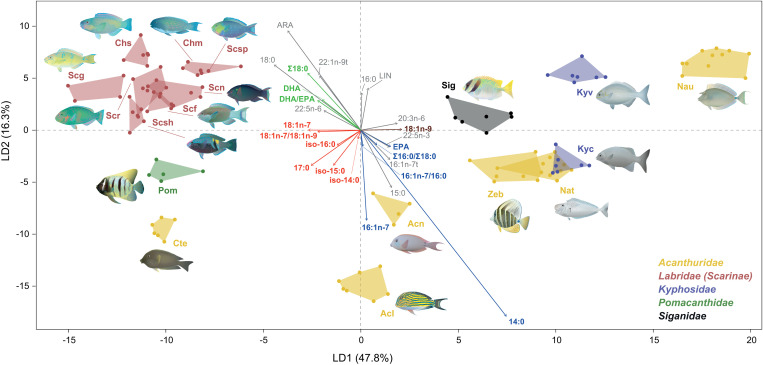
Linear discriminant analysis (LDA) plot illustrating variations in fatty acid (FA) composition (42 FA, values as % relative to total FA) among fish species. LD1 accounts for 47.8% of the variation, and LD2 explains 16.3%. Vectors show the FA that contribute most to group differences (SIMPER) and other FA trophic markers (FATM) indicative of specific dietary sources for the fish. In the vectors, colors other than grey highlight key FATM associated with diatoms (blue), dinoflagellates (green), cyanobacteria (red), and brown algae (brown). Note that 18:1n-7 is also associated with dinoflagellates, and DHA is associated with consumption of invertebrates. For FA abbreviations, refer to S3 Table. Fish families are indicated by color. Acl, *Acanthurus lineatus*, Acn: *A. nigrofuscus*, Cte: *Ctenochaetus striatus*, Nat: *Naso tonganus*, Nau: *N. unicornis*, Zeb: *Zebrasoma velifer*, Kyc: *Kyphosus cinerascens*, Kyv: *K. vaigiensis*, Pom: *Pomacanthus sexstriatus*, Chm: *Chlorurus microrhinos*, Chs: *C. spilurus*, Scf: *Scarus frenatus*, Scg: *S. ghobban*, Scn: *S. niger*, Scr: *S. rivulatus*, Scsh: *S. schlegeli*, Scsp: *S. spinus*, Sig: *Siganus doliatus*.

The LDA also revealed distinct FA composition patterns among fish species (F_17,94 _= 10.73, *p* < 0.001, PERMANOVA; Fig 4). The first linear discriminant component (LD1) clearly separated parrotfishes (Labridae), *P. sexstriatus*, and *C. striatus* from kyphosid chubs, *S. doliatus*, and (other) acanthurids. The second component (LD2) further distinguished parrotfishes from other groups, although they overlapped with *S. doliatus*, *K. vaigiensis*, and *N. unicornis*. SIMPER analysis identified 10 FA (14:0, ARA, 16:0, 16:1n-7, 18:0, 15:0, EPA, DHA, 17:0, and 16:1n-7t, in this order) as the main contributors (>70%) to the observed differences in the LDA (32% overall average dissimilarity among groups). Parrotfishes were mainly associated with ARA, 18:0, 18:1n-7, 22:1n-9t, DHA, and 22:5n-6, but negatively associated with 14:0, 15:0, 20:1n-9, 20:3n-6, 16:1n-7t, 22:5n-3, and EPA, which were positively linked to acanthurids. *Naso unicornis* differed from other acanthurids by being positively associated with LIN. Fatty acids associated with *C. striatus* and *P. sexstriatus* included 17:0 and the BrFA iso-14:0, iso-15:0, and iso-16:0.

**Fig 5:**
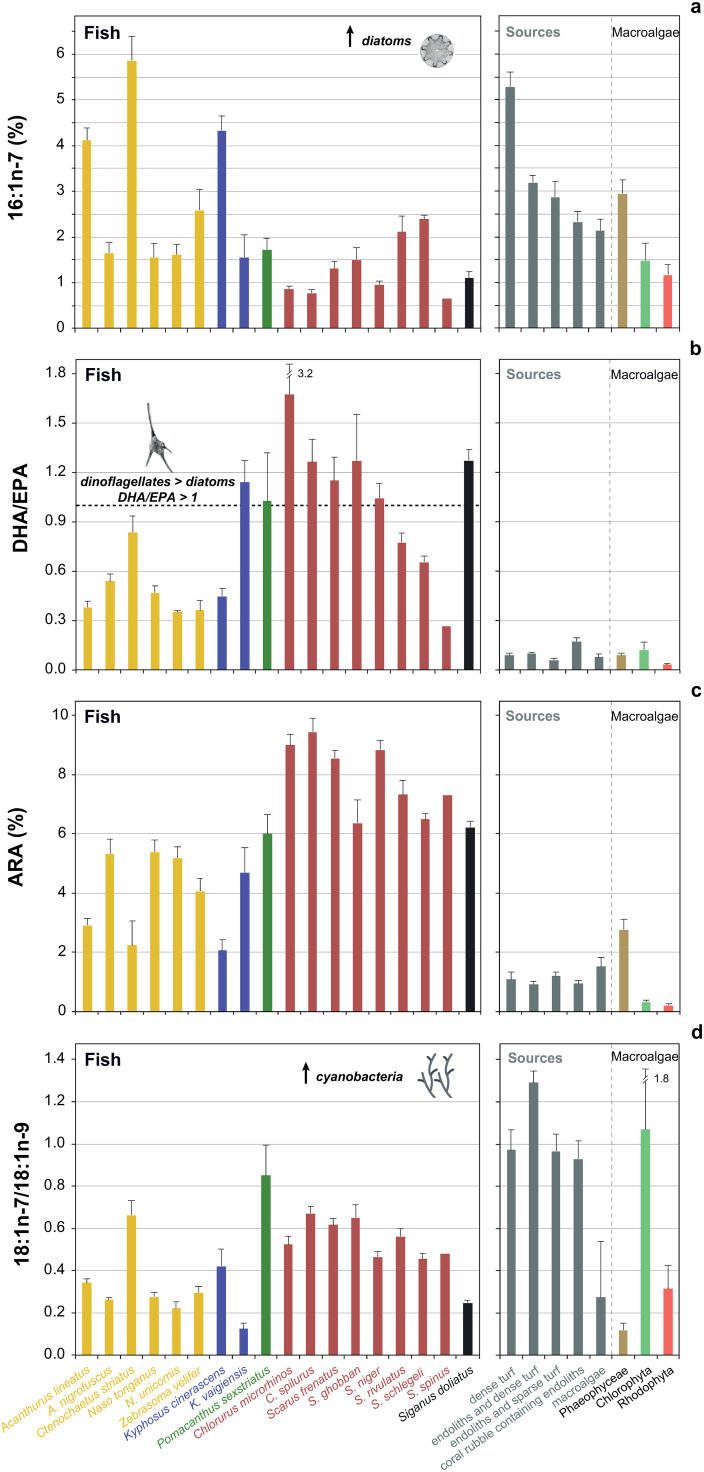
Relative proportion of fatty acid biomarkers across fish species and organic matter sources: a) 16:1n-7 (%), as an indicator for diatoms; b) the DHA/EPA ratio, indicative of dinoflagellate or diatom dominance; c) ARA (%); and d) the 18:1n-7/18:1n-9 ratio, a biomarker for bacteria/cyanobacteria. Fish families are indicated by color: yellow (Acanthuridae), blue (Kyphosidae), green (Pomacanthidae), pink (Labridae–Scarinae), and black (Siganidae). Sources include dense turf, endoliths and dense turf, endoliths and sparse turf, coral rubble containing endoliths, and macroalgae. Macroalgae groups are further categorized by color: brown (Phaeophyceae), light green (Chlorophyta), and red (Rhodophyta). For FA abbreviations, refer to S3 Table.

Important FATM indicative of diatoms (14:0, 16:1n-7, and 16:1n-7/16:0) were found at higher proportions in acanthurids (particularly *C. striatus* and *A. lineatus*) and *K. cinerascens* compared to other groups (Fig 4, Fig 5a, S4 Table). Dinoflagellate indicators (18:1n-7, Σ18:0, DHA, and DHA/EPA > 1) were more abundant in parrotfishes (Fig 5b), whereas cyanobacteria indicators showed the highest values in parrotfishes (18:1n-7 and BrFA), *P. sexstriatus* (17:0, 18:1n-7, and BrFA) and *C. striatus* (18:1n-7 and BrFA) (Fig 4, S4 Table). Note that ARA (Fig 5c) and DHA could also indicate invertebrate consumption in parrotfishes, especially in *Chlorurus* spp. [35,77]. Furthermore, the 18:1n-7/18:1n-9 ratio, an indicator of bacteria/cyanobacteria input, was positively correlated with parrotfishes, *P. sexstriatus*, and *C. striatus* (Fig 5d). The Phaeophyceae FA marker (18:1n-9) was most strongly associated with *N. unicornis*. Table 1 provides a detailed summary of the FATM identified in our fishes and their corresponding dietary sources based on published literature. Finally, significant positive correlations were observed between δ^13^C and LIN (Spearman’s ρ = 0.48, *p* < 0.001), as well as between δ^15^N and iso-14:0 (Spearman’s ρ = 0.41, *p* < 0.001), iso-15:0 (Spearman’s ρ = 0.38, *p* < 0.05), and 21:0 (Spearman’s ρ = 0.40, *p* < 0.05). No other significant correlations were found between isotopic variables and FATM.

**Table 1:** Summary of fatty acid trophic markers (FATM) identified in the fish species investigated in this study and their associated dietary sources based on published literature. For FA abbreviations, refer to S3 Table. The complete list of references is available in the S2 Appendix.

Source	FATM	References
Diatoms	14:0	[1, 2, 3, 4, 5, 6, 7, 8]
	16:1n-7	
	16:1n-7/16:0	
	Σ16:0/Σ18:0	
	EPA	
	DHA/EPA < 1	
Dinoflagellates	18:1n-7	[2, 5, 7, 8, 9, 10]
	Σ18:0	
	DHA	
	DHA/EPA > 1	
Bacteria (cyanobacteria)	17:0	[3, 5, 10, 11, 12, 13, 14]
	18:1n-7	
	18:1n-7/18:1n-9	
	BrFA	
Brown macroalgae (Phaeophyceae)	18:1n-9	[15, 16]

^1^Claustre et al., 1988/1989; ^2^Viso and Marty, 1993; ^3^Kharlamenko et al., 1995; ^4^Napolitano et al., 1997; ^5^Budge and Parrish, 1998; ^6^Budge et al., 2001; ^7^Dalsgaard et al., 2003; ^8^Jónasdóttir et al., 2019; ^9^Nichols et al., 1984; ^10^Sargent et al., 1987; ^11^Perry et al., 1979; ^12^Volkman et al., 1980; ^13^Harvey, 1994; ^14^Yang et al., 2016; ^15^Khotimchenko et al., 2002; ^16^Alfaro et al., 2006.

### Results from mixing models

**Fig 6:**
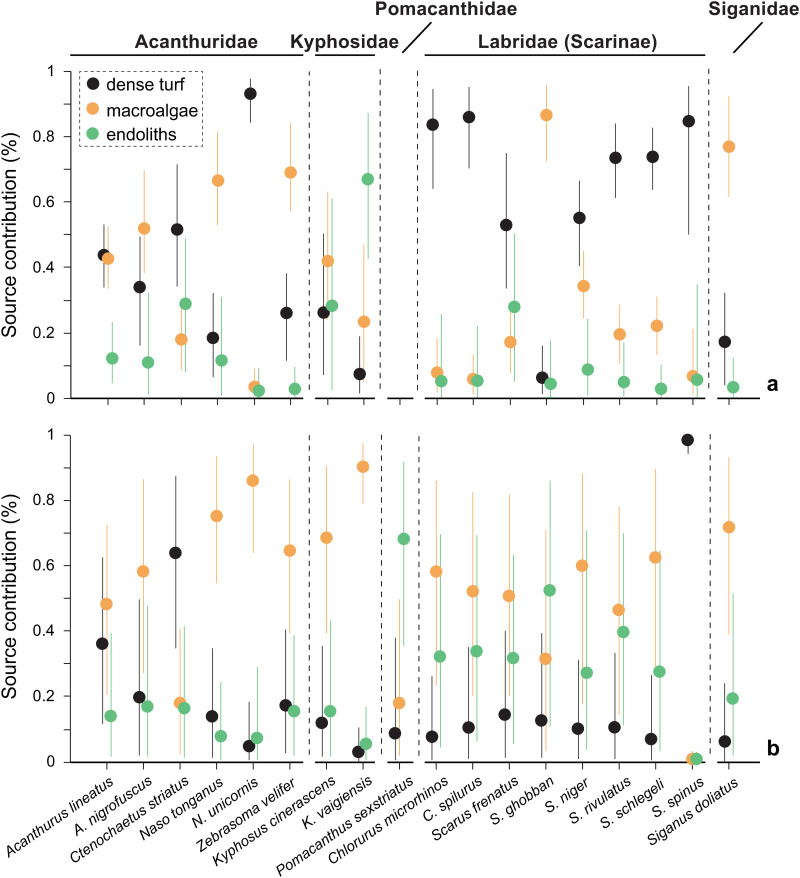
Comparison of source contributions (%) estimated using MixSIAR (median values with 90% Bayesian credible intervals) for the fish species based on: a) the δ^15^N and δ^13^C values, and b) the relative abundance of 42 fatty acids (FA) in the fish muscle tissue. Vertical dashed lines separate different fish families. The models considered three sources: dense turf, macroalgae, and endoliths. In the isotope models, endoliths pooled endoliths and dense turf + endoliths and sparse turf sources, while in the FA models, endoliths included endoliths and dense turf + endoliths and sparse turf + coral rubble containing endoliths. Note that in panel a, source contributions for *Pomacanthus sexstriatus* are not shown, as a logical model could not be computed using stable isotope data for this species (see Results section). Raw data are provided in S5 and S6 Tables.

Mixing models based on SI and FA yielded a different broad picture of the basal source utilization by the fish, especially for parrotfishes, although some similar patterns were identified across other fish families (Fig 6, S5 and S6 Tables). For instance, both approaches indicated that macroalgae are important food sources for most acanthurids (contributing between 45% in *A. lineatus* and 70% in *N. tonganus*), *K. cinerascens* (40–70%), and *S. doliatus* (70%). The two methods also pointed to dense turf as a major component of *S. spinus*’ diet (>85%) and, to a lesser extent, an important source for *C. striatus* (50–60%) and *A. lineatus* (40%). Accordingly, we found a significant correlation between the source proportions estimated using both tracers for these groups (excluding *N. unicornis*; Spearman’s ρ = 0.78, *p* < 0.001). Both models also agreed that endoliths was the least important source (<17%) for acanthurids, except for *C. striatus*. The greatest discrepancies between models were observed in *N. unicornis*, *K. vaigiensis*, and the parrotfishes. According to the isotope models, dense turf was the main source for *N. unicornis* and parrotfishes (except *S. ghobban*), whereas FA models identified macroalgae as the dominant dietary source for these groups. In *K. vaigiensis*, FA models identified macroalgae as the primary source, while isotope models suggested a mixed diet dominated by endoliths. The FA models also assigned greater importance to endoliths for most parrotfishes compared to isotope models, which estimated its contribution as almost negligible. Furthermore, while estimates between the two approaches did not converge for *S. ghobban*, both models indicated that resource use in this species differed from other parrotfishes. Finally, the FA model suggested that *P. sexstriatus*’ diet relied heavily on endolithic sources (68%), more than any other fish species. Overall, isotope models produced more constrained source distributions compared to FA models.

## Discussion

It is well established that SI and FA composition in fish tissues reflects the assimilation of dietary inputs and can effectively inform the feeding ecology and trophic relationships of marine fishes [32,78]. Our study sought to clarify and define patterns of resource use among various taxa of roving herbivorous coral-reef fishes. Our findings indicate that trophic partitioning among these groups is more pronounced than previously recognized, revealing distinct feeding strategies among algivores, detritivorous surgeonfishes, and parrotfishes, the latter exhibiting uniquely complex diets that depend on microscopic photoautotrophs including cyanobacteria and dinoflagellates. To our knowledge, this is the first study to integrate SI and FA analyses to investigate the trophic ecology of a diverse assemblage of co-occurring reef fish species from the Great Barrier Reef.

### Isotopic composition of the organic matter sources

The isotopic composition of the potential organic matter sources for these fishes was within the common range observed in previous studies [79,80]. These sources showed large variability in δ^13^C values, leading to a high degree of overlap between macroalgae and other groups (S2 Table). For example, the isotopic composition of brown algae resembled that of endoliths and dense turf, endoliths and sparse turf, and dense turf sources, whereas green and red macroalgal species were significantly more depleted in ^13^C. The latter showed an isotopic composition overlapping with that of coral rubble containing endoliths. This result is consistent with the observations of Mill and colleagues [72], who evaluated the isotopic composition of several macroalgal species from a reef in Oman and also found that *Turbinaria* was less depleted in ^13^C compared to other brown, red, and green algae. At Lizard Island, *Turbinaria* is often covered with cyanobacterial epiphytes [10], which are typically enriched in ^13^C relative to other aquatic autotrophs [81], potentially increasing the δ^13^C value observed in *Turbinaria*. In general, macroalgae exhibit a high degree of variability in δ^13^C values [82,83], particularly red algae, for which two groups have been defined in the literature: i) subtidal or shaded intertidal red algae with δ^13^C values ≤ −30‰, likely utilizing CO_2_, and ii) other red algae with δ^13^C values ranging from −30 to −10‰. Red algae consistently display lower δ^13^C values compared to green and brown algae. Moreover, the C:N observed in most macroalgae (C:N > 10) indicate a lower nutritional quality of these sources relative to other organic matter types studied (e.g., [80]). The C:N values of dense turf, and the three endolithic categories (C:N ~ 7) are comparable to those reported for marine particulate organic matter in the tropical coral ecosystems of the Marquesas Islands [35], and closely align with the values for diatoms and cyanobacteria (C:N = 5.9 and 5.2, respectively) [84], suggesting that these sources may be relatively more nutritionally valuable to fish.

### Isotopic composition of the fishes

The stable C and N isotopic signatures effectively differentiated fish families, revealing different functional groups with characteristic nutritional strategies. Based on δ^15^N values, the angelfish *P. sexstriatus* in our study sat almost one trophic position (TP) above most other fish groups examined, especially parrotfishes, with δ^15^N ~ 3.3‰ higher [73]. The diet composition of *P. sexstriatus* at our study sites is unknown, but it is thought to eat a mixture of epilithic sponges and algae [85,86], leading to higher ^15^N enrichment compared to strictly herbivorous species [35,87]. The herbivorous *K. vaigiensis*, which browses brown macroalgae such as *Turbinaria* sp. and *Dictyota* sp. [10], displayed relatively high δ^15^N values compared to other algivores. This likely reflects nitrogen recycling in the gut by hindgut symbionts [88–90], although the precise mechanisms remain unclear (see [91,92]). Conversely, parrotfishes exhibited the highest δ^13^C values and the lowest δ^15^N values in our dataset, suggesting a lower TP than the other fish examined. Similar isotopic differences between parrotfishes and other co-occurring herbivorous fish taxa, such as acanthurids, siganids, and kyphosid chubs, have been consistently reported across reef and seagrass habitats (e.g., [9,12,93]), revealing complex trophic ecologies and a high level of functional diversity.

The isotopic baseline of the sources sampled along with the fishes suggests that parrotfishes primarily feed on turf, particularly dense turf, which exhibited the lowest δ^15^N values (also comparatively high δ^13^C values) among our source samples. Results from mixing models further support a major input of dense turf to the diets of parrotfishes, except for *S. ghobban*. These findings agree with those of Carassou and colleagues [93], who examined the feeding habits of several parrotfish species from New Caledonia and observed a positive association between depleted ^15^N and enriched ^13^C signatures in parrotfishes feeding on turf and detrital material. Clements and colleagues [12] suggested that the characteristic low δ^15^N values of grazing parrotfishes may reflect a dietary target of protein-rich diazotrophic microphotoautotrophs, i.e., filamentous cyanobacteria found among turf. Diazotrophs, such as N_2_-fixing cyanobacteria, display lower δ^15^N and higher δ^13^C values than other aquatic autotrophs [37,39]. *Acanthurus nigrofuscus*, *A. lineatus*, *N. tonganus*, and *K. cinerascens* showed isotopic compositions slightly enriched in ^15^N and depleted in ^13^C compared to most parrotfish (except *S. ghobban*). The two *Acanthurus* species primarily graze on red and green filamentous turf algae, whereas *N. tonganus* and *K. cinerascens* predominantly target red and green macroalgae [10,94,95]. The muscle isotopic composition of *Z. velifer* closely resembles that of *S. doliatus*, both of which target red and green algal turf and macroalgae [11,94]. The relatively low δ^15^N signal observed in parrotfishes may indicate the assimilation of diazotrophic filamentous cyanobacteria within turf or as epiphytes on macroalgae [12].

The isotopic data also indicated resource partitioning across species within the same family. *Scarus ghobban* displayed high trophic niche segregation, with the highest δ^15^N values among parrotfishes. Plass-Johnson and colleagues [36] observed that the enriched ^15^N signal of this species distinguished it from other scraping parrotfishes, which they attributed to its earlier developmental stage. However, differences in δ^15^N values between *S. ghobban* individuals in the initial phase (equivalent to females) and other scraping parrotfishes in the terminal phase (equivalent to males) were not significant. In our dataset, *S. ghobban* specimens were classified as initial phase, while the other parrotfish species were classified as terminal phase. We hypothesize that, rather than reflecting sex-related differences between *S. ghobban* and the other parrotfishes, the observed isotopic differences may be explained by *S. ghobban* targeting microscopic photoautotrophs that are endosymbionts or possibly filtered prey of sessile invertebrates such as sponges and tunicates [96–98], resulting in an increased δ^15^N signal [36]. Our data also support the dietary dichotomy observed by [36] between excavators (i.e., *Chlorurus* spp., with higher δ^15^N) and scrapers (i.e., *Scarus* spp., with lower δ^15^N, excepting S. *ghobban*) [99]. Excavating species, such as *C. microrhinos*, likely ingest a lower proportion of diazotrophic cyanobacteria than scraping *Scarus* species, and access endolithic material, which may include the microscopic chlorophyte *Ostreobium* spp. and endolithic sponges [16,17]. This likely enriches the δ^15^N signal of excavators compared to *Scarus* species. *Scarus spinus* exhibited the highest δ^13^C values among parrotfishes, suggesting a stronger δ^13^C signal from cyanobacteria. This species has a highly specialized diet consisting of the epilithic and superficial endolithic protein-rich microphotoautotrophs, particularly filamentous cyanobacteria associated with crustose coralline algae (CCA) [100].

Two acanthurid species deviated isotopically from other members of their family: *C. striatus* (in terms of δ^13^C) and *N. unicornis* (in terms of both δ^13^C and δ^15^N). Mixing model results indicated a major proportion of dense turf in the diet of *C. striatus*, in contrast to the large input of macroalgae in the diets of other acanthurids. *Ctenochaetus striatus* has long been recognized as a detritivorous fish that ‘brushes’ detritus off algal turf and other reef surfaces, as demonstrated by observational and morphological studies [10,18,95]. However, the composition of this detritus has been uncertain due to mechanical trituration in the gizzard. Recent work using essential amino acid δ^13^C fingerprints and sea cucumbers as end-member sources suggested detrital microalgae as a dominant source of dietary amino acids [34], while diet metabarcoding indicated a broader range of ingested items, predominantly dinoflagellates [9]. In our study, *N. unicornis* fed predominantly on the brown alga *T. conoides*, which is often covered with cyanobacterial epiphytes. However, isotope mixing models identified dense turf as the main source for *N. unicornis*. The combined inputs of enriched ^13^C from protein-poor *T. conoides* and depleted ^15^N from protein-rich cyanobacterial epiphytes may significantly shape the overall isotopic profile of *N. unicornis*.

### Fatty acid profiles of the organic matter sources

This study represents the most extensive assessment to date of FA composition in marine herbivorous fish and their food sources. First, we sought to identify specific FA that could unambiguously differentiate each source and trace them into their primary consumers. Our analysis revealed that the FA profiles of the selected organic matter sources (i.e., dense turf, macroalgae, and endolithic sources) were significantly distinct; however, no single FA was exclusively associated with any particular source category. Nonetheless, we identified significant contributions of certain FATM (Table 1) that could be linked to specific producers. We found strong evidence of diatom contribution to dense turf, supported by high % of 14:0, 16:1n-7, and EPA, whereas high % of 18:1n-7 and BrFA indicated a cyanobacterial contribution to endoliths [29,38]. The latter reflects the known abundance of cyanobacteria in euendolithic communities [47,101]. A high contribution of EPA was also reported in turf algae from the coral reef ecosystem of the Marquesas Islands [80]. Both dense turf and endolithic source categories include epilithic algal turfs, which host a diversity of microscopic autotrophic organisms, including diatoms and cyanobacteria [23]. However, endolithic sources consist of both epilithic algal turf and the underlying euendolithic biota [12]. Within the macroalgae category, we also found differences between brown algae and other macroalgal taxa. Brown algae exhibited higher % of 18:1n-9, LIN, and ARA compared to other sampled algal or organic matter sources. While 18:1n-9 is abundant in brown algae [102–104], ARA has been widely recognized as a particular trait of red algae [80,102,105], and is usually found in lower proportions in brown algae [38,106,107].

### Fatty acid composition of the fishes

Although not yet extensively studied, it is well recognized that tropical fishes have distinct FA profiles compared to temperate and polar fishes, and also that the FA composition differs between herbivorous and carnivorous fish [77]. Long chain PUFA, especially EPA and DHA, are less abundant in tropical marine fishes, whereas ARA shows the opposite trend. Moreover, DHA levels tend to increase with TP in marine fishes, while ARA levels show the opposite trend [77,108]. The fish from the Great Barrier Reef ecosystem contained low proportions of EPA (<3.9%) and DHA (<2.8%), but relatively high ARA levels (2.1–9.5%), similar to those found in tropical herbivorous fish from other regions (e.g., [35,109]). Our findings demonstrated that parrotfishes, kyphosid chubs, acanthurid surgeonfishes, and unicornfishes, as well as the single studied species of angelfish *P. sexstriatus* and rabbitfish *S. doliatus* grouped based on FA composition, aligning with closely related and ecologically similar species, although with some exceptions. Individual species or genera could be further distinguished within these groups. Differences in FA profiles are thought to primarily reflect dietary variations rather than phylogenetic relationship [109]. Our results support the idea that reef fishes are selective feeders that partition photoautotrophic resources [12,110].

Overall, the separation of fish groups based on FA profiles largely corresponded with groupings derived from isotopic data. Notable similarities between these two biomarker datasets included: i) *P. sexstriatus* being a distinct group, ii) kyphosid chubs, particularly *K. cinerascens*, clustering closely to acanthurids, iii) *S. doliatus* being closely related to *Z. velifer*, iv) *N. unicornis* and *C. striatus* each forming distinct groups separate from other acanthurids, v) the two sampled *Acanthurus* species grouping closely together, and vi) *Z. velifer* and *N. tonganus* being closely related. Nevertheless, some differences were apparent between biomarkers. For example, *S. ghobban* grouped with other parrotfishes based on FA composition but was distinct from them based on SI composition. The FA profiles provided better separation between parrotfishes and acanthurids, whereas isotopic signatures of these groups showed some overlap. Within parrotfishes, *Chlorurus* and *Scarus* species were more clearly separated based on FA composition.

### Fatty acid trophic markers identified in the fish species

The relative proportions of individual FATM in the fish allowed to identify the contribution of distinctive sources to their diet (Table 1). We found high levels of FATM associated with diatoms, i.e., 14:0, 16:1n-7, 16:1n-7/16:0, and Σ16:0/Σ18:0 [29,38] in the surgeonfishes *C. striatus* and *A. lineatus* and in *K. cinerascens*. In contrast, low levels of diatom markers were found in parrotfishes. A study in the Northwestern Hawaiian Islands [109] also found that detritivorous surgeonfishes such as *Acanthurus olivaceus* and *Ctenochaetus strigosus* contain higher proportions of 16:1n-7 than parrotfishes. The abundance of this FA has also been associated with diatom consumption in other surgeonfishes, including *A. nigricans* and *C. marginatus* from the Marquesas Islands [35].

C18 FA, and especially > 20C PUFA, are characteristic of dinoflagellates [29]. High levels of dinoflagellate FATM such as 18:1n-7, 18:1n-7/18:1n-9, and DHA [29,63,111] were associated with our parrotfishes. The ratio DHA/EPA > 1 revealed that dinoflagellates generally dominated over diatoms in the diet of *K. vaigiensis*, *P. sexstriatus*, *Chlorurus*, and most *Scarus* species, as well as *S. doliatus*, and the reverse in acanthurids and *K. cinerascens*. Notably, *S. spinus* exhibited a lower DHA/EPA ratio (0.3), suggesting a greater input of diatoms compared to other parrotfishes, with DHA/EPA usually >1 [100]. A less abundant FA (<1% relative abundance) that exhibited the highest values in parrotfishes was 22:5n-6. Piché and colleagues [109] observed that this scarce FA was one of the fifteen with the highest variance across reef fishes and invertebrates from the Northwestern Hawaiian Islands. Likewise, high levels of 22:5n-6 were associated with *Scarus* spp. when compared with other nominally herbivorous species such as *A. nigricans*, and even higher trophic level consumers, including zooplankton and filter-feeders [35]. Although 22:5n-6 has not been definitively attributed to a specific dietary source, Dalsgaard and colleagues [29] noted that elevated levels of C22 PUFA are usually indicative of dinoflagellates. Therefore, our findings suggest that most *Scarus* and *Chlorurus* species directly or indirectly ingest dinoflagellates.

Bacteria also biosynthesize many of the common FA present in phytoplankton, including FATM of dinoflagellates such as 18:1n-7 (e.g., [112]), making it challenging to distinguish bacterial vs eukaryotic contributions to fish diets. Unless elevated levels of specific bacterial markers such as BrFA [113] are also detected, it is generally safer to assume that these FA derive from eukaryotic production [29]. Our data showed that parrotfishes are characterized by high abundance not only of 18:1n-7 and DHA, but also of other indicators of bacteria, such as 18:1n-7/18:1n-9 (in general for all parrotfishes), 17:0, and BrFA (in *S. ghobban*) [38,114]. Piché and colleagues [109] also found that parrotfishes are clearly distinct from other herbivorous fish by having high values of 18:1n-7. BrFA are common constituents of bacteria, yet rarely detected in other microorganisms [113]. Bacterial signal (especially the BrFA iso-17:0) has been detected in primary consumers from Marquesas Islands including parrotfishes and acanthurids, demonstrating that bacteria contribute to the organic matter sources supporting reef food webs [35]. Recent studies using rRNA metabarcoding of bite cores and pharyngeal contents have demonstrated the importance of cyanobacteria in the diet of Indo-Pacific parrotfishes [9,13,15,16]. Overall, our findings suggest strong contribution of dinoflagellates to the diet of parrotfishes, but also that the contribution of bacteria (most importantly to *S. ghobban*) cannot be ignored. This is also supported by the SI composition of C and N in the fish samples. Other species besides parrotfishes also exhibited high levels of BrFA, in some cases exceeding those in *S. ghobban*, notably *C. striatus* and *P. sexstriatus*. This suggests a stronger contribution of bacterial FA to their diets. It is most likely that *P. sexstriatus* and *S. ghobban* eat epilithic sponges [86] that contain bacterial symbionts, including potentially cyanobacteria. This would also explain why these species exhibited the highest δ^15^N values, the highest contribution of endolithic sources in FA mixing models (as endolithic sources presented more cyanobacteria-related FA than dense turf and macroalgae), and the highest contributions of bacterial FATM.

Parrotfishes displayed the highest levels of ARA and DHA, with ARA being particularly abundant in *Chlorurus* spp. and DHA most prevalent in *C. microrhinos* and *S. ghobban*. As mentioned above, high values of ARA were also found in Hawaiian parrotfishes by Piché and colleagues [109], and in two *Scarus* spp. by Fey and colleagues [35]. ARA is abundant in sponges, CCA, and coral mucus [115], while DHA is a biomarker for dinoflagellates [38,116]. CCA is a dominant benthic component of the feeding substrata targeted by parrotfishes [14], while coral mucus is an important component of particulate organic matter on coral reefs [117] and serves as a crucial source of nutrients for sponges and benthic microbes [118]. The elevated levels of ARA and DHA in parrotfishes are therefore consistent with the hypothesis that these parrotfishes are microphages that target dinoflagellates and other protein-rich photoautotrophic microorganisms that live on (epilithic) or within (endolithic) calcareous substrata, are epiphytic on algae or seagrasses, or endosymbionts of sessile invertebrates such as sponges and tunicates [12]. Opisthobranch molluscs also target sessile invertebrates for their photoautotrophic endosymbionts [96], while filter-feeding sponges and tunicates concentrate phytoplankton and non-photoautotrophic picoplankton through filter-feeding [97,98]. ARA has also been suggested as a biomarker for macroalgae [80,102]. Among our dietary source samples, brown algae exhibited the highest ARA levels, suggesting they may contribute to the parrotfish diet. However, 18:1n-9 is also characteristic of brown algae, yet no important signal of this FA was detected in parrotfishes. This discrepancy suggests that ARA in parrotfishes originates from other food resources. Furthermore, there is little evidence supporting the idea that *Chlorurus* and *Scarus* species target brown algae. The storage carbohydrates of brown algae (excluding Dictyotales, which are unusual in storing energy as lipid; [119]) are largely indigestible to parrotfishes [12], and brown macroalgae are minor components on the feeding substrata of the study parrotfish species [13,16].

Only the sampled primary producer sources were considered as potential dietary sources for the fish in the mixing models, and so caution is required in interpreting the model outcomes. The FA models identified macroalgae as the main source for parrotfishes, especially in *Chlorurus* spp., likely due to the high ARA levels detected in both macroalgae and parrotfishes. The chlorophyte *Ostreobium* is one of the most common genera of autotrophic euendoliths in coral reef substrates worldwide [101], including carbonate sediment [120]. This microscopic green alga is likely an important dietary source for excavating and some scraping parrotfishes [12]. Massé and colleagues [121] observed that free-living forms of *Ostreobium* exhibit high ARA levels, in contrast to endolithic forms. They also found that 18:1n-7 was abundant in both *Ostreobium* forms. Thus, the presence of ARA and 18:1n-7 could potentially be a signal of *Ostreobium* in *Chlorurus* species. Additionally, we detected high levels of 18:1n-9, a biomarker of brown algae [103,104] in acanthurids, but especially in *N. unicornis*. High levels of this FA, along with LIN and ARA, were responsible for distinguishing brown algae from the other algal groups and endmembers studied. *Naso unicornis* also exhibited the highest LIN levels among all studied fish species, further supporting its reliance on a brown algae-based diet. Similarly, relatively high levels of these FA were found in *K. vaigiensis*, a species known to primarily consume brown macroalgae [10,122]. These findings underscore the importance of 18:1n-9, LIN, and ARA in tracing the contribution of brown algae to reef food webs. Our SI and FA biomarker data align with previous gut content analyses indicating that *N. unicornis* primarily feeds on brown and green macroalgae [10,110]. These include taxa such as *Sargassum* and *Turbinaria*, that can carry high epiphyte loads [49,50]. As suggested earlier, the assimilation of depleted ^15^N from cyanobacterial epiphytes likely explains the relatively low δ^15^N signal observed in *N. unicornis*. Results from MixSIAR FA models also support the expected large dietary input of macroalgae to *N. unicornis* and *K. vaigiensis*.

### Trophic discrimination of nitrogen in herbivorous fishes

To accurately account for the trophic shift in δ^15^N values used in MixSIAR models, the δ^15^N TDF must be multiplied by the number of trophic levels separating the consumer and the basal sources [25]. From an ecological perspective, herbivorous fish are considered primary consumers (TP = 2), so the shift in δ^15^N values between them and their food sources (equivalent to a 1 TP difference) should be a reasonable proxy for their actual δ^15^N TDF. Based on this principle, we estimated appropriate TDF using the difference between the δ^15^N values of the target fish consumers and the basal diet sources. The study fish families differed in δ^15^N TDF values: ~ 3‰ for parrotfishes, 4‰ for acanthurids and siganids, 4.5‰ for kyphosid chubs, and 5‰ for pomacanthids. These values are similar to those previously reported for herbivorous fishes (e.g., [72, 93, 123]), and suggest that δ^15^N TDF values for herbivorous fishes are markedly higher than the widely accepted 3.4‰ value [73]. Different meta-analyses (e.g., [124–126]) have identified a negative relationship between a fish’s TP (estimated from bulk δ^15^N values) and the δ^15^N TDF, with herbivores having higher TDF than omnivores and carnivores. In contrast, Wyatt and colleagues [127] observed that the δ^15^N TDF increased with TP in coral reef fishes from Western Australia, resulting in carnivores displaying higher δ^15^N TDF than herbivores. Regardless of the direction of this relationship, an accurate estimation of diets and characterization of trophic interactions in reef ecosystems requires the use of species-specific rather than generalized TDF values. The reasons driving the differences in δ^15^N TDF among fishes with distinct feeding strategies remain unclear. The trophic ecology of herbivores and carnivores differs in terms of diet quality (e.g., C:N ratio) and food processing mechanisms, among other factors [91]. Unlike carnivorous fish, the high δ^15^N TDF observed in herbivorous fish has been associated with poor quality diets (i.e., low protein content and high C:N), greater de novo synthesis of non-essential amino acids, and high consumption and excretion rates [72,126].

### Comparison of mixing model approaches

Few studies have evaluated the consistency and reliability of different biomarkers to predict diet in the same consumer species. In this study, we assessed the overall agreement between SI and FA tracers to estimate basal dietary sources for fish in the Great Barrier Reef using Bayesian mixing models. Based on previous observations in the literature [27], we expected these models to produce similar results. In a review of dietary tracing methods, Nielsen and colleagues [78] reported that SI (δ^13^C and δ^15^N) and FA approaches typically show good agreement, with a Czekanowski’s similarity index of ~80%, particularly when the number of potential food sources is limited (<3). However, our findings indicate that an overall agreement between the two methods was not always achieved, especially for parrotfishes. The FA estimated source proportions provided better differentiation of major fish groups based on expected diet and ecology. A recent study by Pickett and colleagues [28] demonstrated that Bayesian mixing models using FA gave the most accurate diet estimates in two freshwater fishes, compared to amino acids, and to SI (δ^13^C and δ^15^N) models that did not converge. Diets that include a greater number of basal sources are likely to result in limited discriminating power relative to the number of tracers (e.g., only two for SI, [128]). More accurate diet estimates can often be achieved by incorporating additional tracers, such as FA [129]. However, when comparing different biomarker methods, such as SI and FA, it is crucial to account for the different temporal scales over which they integrate dietary information, as nutrient turnover rates vary between tissues [130]. Besides, using two isotope tracers typically allows differentiation of only three to five predefined dietary groups [25], whereas FA-based methods can increase the level of dietary resolution as the number of tracers included grows [28]. The FATM indicated that some of the fish species, such as *P. sexstriatus*, receive food inputs from consumer organisms. This finding explains why isotope tracers failed to reliably determine contributions from specific sources for this species. As noted by Nielsen and colleagues [78], prior knowledge of the consumer’s feeding ecology and the available resources in its environment improves the accuracy of dietary assessments. However, determining potential resources is difficult for grazing taxa such as parrotfishes, which ingest complex mixtures of food items. In such cases, sampling the complete set of end member sources requires prior knowledge of the diet itself, posing a fundamental challenge to dietary analysis.

## Conclusions

While the isotopic and FA biomarker and modelling analyses in this study were not definitive for delineating the diets of several fish species, they demonstrated a level of trophic partitioning among the study species that is generally overlooked in the literature and is comparable to that observed in freshwater grazing fish assemblages. Our isotope and FA analyses broadly supported previous gut content data on algivorous acanthurid species, the rabbitfish *S. doliatus*, and the two *Kyphosus* species. Caveats to this include *N. unicornis*, which appeared to obtain significant levels of dietary protein from epiphytic cyanobacteria despite a diet dominated by macroalgae, and *K. vaigiensis*, in which dietary signal may be distorted by the importance of gut microbiota in transforming dietary components. The detritivorous *C. striatus* was isotopically similar to parrotfishes but distinct in terms of FA, likely due to a greater input of diatoms. This finding supports previous research showing that *C. striatus* and *A. lineatus*, despite sharing feeding substrata, exhibit different feeding behavior and ingest different resources [18,131]. The results also reinforce the distinctive nature of parrotfish diets, with a focus on protein-rich microscopic photoautotrophs, especially cyanobacteria and dinoflagellates, harvested from a variety of different substrata [12–14,16,17]. The importance of dinoflagellates in parrotfish diets is also supported by diet metabarcoding data [9,15]. Some parrotfish species, such as *S. ghobban*, clearly target sessile invertebrates such as sponges, although perhaps because these are a concentrated source of microscopic photoautotrophs including both cyanobacteria and dinoflagellates. The diets of parrotfishes are highly complex and poorly understood for most species, yet they are clearly distinct from those of algivorous and detritivorous surgeonfishes. Overall, this study highlighted the importance of using multiple approaches to characterize the trophic ecology of herbivorous coral reef fishes, particularly because these fishes obtain a balanced macronutrient intake from multiple food sources.

### Acronyms

**Table:** 

FA	fatty acid (s)	ARA	arachidonic acid
FATM	fatty acid trophic marker (s)	EPA	eicosapentaenoic acid
SFA	saturated fatty acid (s)	DHA	docosahexaenoic acid
MUFA	monounsaturated fatty acid (s)	SI	stable isotope (s)
PUFA	polyunsaturated fatty acid (s)	TDF	trophic discrimination factor (s)
BrFA	branched fatty acid (s)	CC	calibration coefficient (s)
LIN	linoleic acid	TP	trophic position
ALA	α-linolenic acid		

## Supporting information

S1 Appendix Supplementary methods. (DOCX)

S2 Appendix Supplementary References for Table 1. (DOCX)

S1 Checklist Additional information regarding the ethical, cultural, and scientific considerations specific to inclusivity in global research. (DOCX)

S1 Dataset Stable isotope and fatty acid data for the fish and the organic matter sources. (XLSX)

S1 Fig Representative *in situ* photographs of endolithic sources. Left: endoliths and dense turf. Right: endoliths and sparse turf.
(TIF)

S2 Fig Linear discriminant analysis (LDA) plot illustrating differences in fatty acid (FA) composition (42 FA, values as % relative to total FA) among the organic matter sources. LD1 explains 96.1% of the variation and LD2 explains 2.1%. Sources are grouped in three clusters (dense turf, macroalgae and endolithic categories) according to their FA composition (PERMANOVA, F_2,41 _= 5.75, *p* < 0.001). Vectors indicate those individual FA contributing most to the overall variance among groups (SIMPER). For FA abbreviations, refer to S3 Table.
(TIF)

S1 Table Fish descriptors: standard length range (SL, length to the end of the vertebral column), fork length range (FL, length including the tail), total weight range (TW, wet weight), sex, and color phases (in parrotfishes). IP (initial phase) is equivalent to female (F) and TP (terminal phase) is equivalent to male (M) except in *Chlorurus microrhinos* and *Scarus niger*, where both sexes are TP.
(DOCX)

S2 Table Mean (± sd) δ^15^N and δ^13^C values (‰) of the organic matter sources and reef fish species collected near the Lizard Island Complex in Australia. Mean C:N ratio values (± sd) are also shown. *P*: Phaeophyceae, *C*: Chlorophyta, *R*: Rhodophyta.
(DOCX)

S3 Table Fatty acids (FA) composition (% of total FA or FA ratios, mean ± se) of the organic matter sources. Background shading indicates the most abundant FA (i.e., 18, average proportions greater than 1% in at least one source type). Different letters indicate significant differences (Dunn’s post hoc after significant Kruskal-Wallis test, *p* < 0.05) between the main five organic matter sources or between the macroalgae groups. For those FA or FA trophic markers displaying significant differences across sources, the highest value is shown in boldface. Data are also shown for individual macroalgae groups. Phaeophyceae pooled data from *Padina boergesenii* and *Turbinaria conoides*, Chlorophyta pooled data from *Dictyosphaeria versluysii* and *Halimeda macrophysa*, and Rhodophyta pooled data from *Galaxaura marginata*, *Ganonema farinosum* and *Portieria hornemannii*. SFA: saturated FA, MUFA: monounsaturated FA, PUFA: polyunsaturated FA, LIN: linoleic acid, ALA: α-linolenic acid, ARA: arachidonic acid, EPA: eicosapentaenoic acid, DHA: docosahexaenoic acid, Σn-3 PUFA: sum of n-3 PUFA; Σn-6 PUFA: sum of n-6 PUFA, Σn-3/Σn-6: sum of n-3 PUFA/sum of n-6 PUFA. Branched FA (BrFA) are iso-14:0, iso-15:0 and iso-16:0.
(DOCX)

S4 Table Fatty acids (FA) composition (% of total FA or FA ratios, mean ± se) in fish muscle tissue. Background shading indicates the most abundant FA (i.e., 21, average proportions greater than 1% in at least one species). Different letters indicate significant differences (Dunn’s post hoc after significant Kruskal-Wallis test, *p* < 0.05) between species. For those FA or FA trophic markers displaying significant differences across species, the highest values are shown in boldface. For FA abbreviations, refer to S3 Table.
(DOCX)

S5 Table Source proportions contributing to the fish diets estimated by stable isotope-based mixing models. The results are shown as the median (50% quartile) and the associated 90% Bayesian credible intervals (BCI) of diet proportions in brackets. Endoliths pooled endoliths and dense turf + endoliths and sparse turf sources. Var.: total variation explained by the model. Highest median contributions for each taxon are shown in bold type. These data are depicted visually in Fig 6a. Note that consumer data lying outside the mixing source polygon (see Fig 3b) were excluded from these models.
(DOCX)

S6 Table Source proportions contributing to the fish diets estimated by fatty acid-based mixing models. The results are shown as the median (50% quartile) and the associated 90% Bayesian credible intervals (BCI) of diet proportions in brackets. Endoliths pooled endoliths and dense turf + endoliths and sparse turf + coral rubble containing endoliths sources. Var.: total variation explained by the model. Highest median contributions for each taxon are shown in bold type. These data are depicted visually in Fig 6b.
(DOCX)
