# Supplementary material for: Characterizing the trophic ecology of herbivorous coral reef fishes using stable isotope and fatty acid biomarkers
Source: PLoS One. 2025 Jun 30;20(6):e0327594. doi: 10.1371/journal.pone.0327594 (PMC12208496; doi:10.1371/journal.pone.0327594)
Supplement: S1 Appendix — (DOCX) [file pone.0327594.s001.docx]

*Fatty acid analysis*

The fatty acid composition of lipids was analyzed in all fish muscle samples (n=111) and in some samples of organic matter sources (n=44): i.e. dense turf (n=3), endoliths and dense turf (n=6), endoliths and sparse turf (n=6), coral rubble containing endoliths (n=3), and macroalgae including *Padina boergesenii* (n=3), *Turbinaria conoides* thallus (n=7), *T. conoides* reproductive tissue (n=3), *Dictyosphaeria versluysii* (n=3), *Halimeda macrophysa* (n=4), *Galaxaura marginata* (n=4), *Ganonema farinosum* (n=1), and *Portieria hornemannii* (n=1).

Briefly, tissue aliquots (~10 mg for fish muscle and ~150 mg for organic matter sources, dry weight) were weighted in Borosilicate glass tubes equipped with Teflon-lined screw-caps. Then, an internal standard solution consisting of 0.083 mg.mL^-1^ of nonadecanoic acid (C_19:0_) and 0.082 mg.mL^-1^ of tridecanoic acid (C_23:0_), dissolved in 2 mL of methanol:benzene (4:l v/v, Analytical Grade, Merck), was added to each sample. A small magnetic stirring bar was placed in each tube and, while stirring, 200 μL of acetyl chloride (ECP Labchem) was added slowly, dropwise to each sample over a 1 min period. Tubes were closed tightly and tape was wrapped around the outside in order to check for potential leakage. The transesterification reaction was carried out in a Reacti-Therm heating and stirring block at 100ºC for 1 h. After cooling in water, 5 mL of 6% K_2_CO_3_ solution was added to each tube to stop the reaction and neutralize the mixture, followed by vortexing and centrifugation (2500 rpm, 5 min at room temperature). The upper benzene phase was recovered, and an aliquot (50% dilution in toluene) was transferred to a GC vial with an insert for analysis by gas chromatography-mass spectrometry (GC-MS). Laboratory controls were included during the transesterification step (i.e. a positive control containing 52 FA reference standards all with different concentrations, and a negative control containing a mixture of the internal standards C_19:0_ and C_23:0_ at the same concentration as in the samples).

After direct transesterification of the samples, the resulting individual fatty acid methyl esters (FAME) were identified and semi-quantified by GC-MS. Briefly, 1 μL of each sample was injected into a 4 mm ID straight glass inlet liner packed with deactivated glass wool (Restek Sky®) using an Agilent 7693 AutoSampler. The inlet temperature was set to 250ºC in splitless mode, with a column flow rate of 3 mL.min^-1^, and a column head pressure of 42.21 psi, giving a total flow of 55 mL.min^-1^. Purge flow was set to 50 mL.min^-1^, activated 1 min after injection. The carrier gas was instrument-grade helium (99.99%, BOC). The GC oven temperature was initially held at 45ºC for 2 min, then increased to 215ºC with a gradient of 10ºC.min^-1^ and held for 35 min. The temperature was then further increased at 40ºC.min^-1^ to 250ºC and held for 10 min. The transfer line to the mass spectrometric detector (MSD) was maintained at 250ºC, with the MSD source at 230ºC and the MSD quadropole at 150ºC. The detector was turned on 12 min into the run and operated in positive-ion, electron-impact ionization mode at 70 eV electron energy, with the electron multiplier set to the autotune value without additional voltage. Data were acquired at 1463 amu/s in scan mode, covering a mass range of 41 to 420 atomic mass units, with a detection threshold of 100 ion counts.
