# Supplementary material for: Characterizing the trophic ecology of herbivorous coral reef fishes using stable isotope and fatty acid biomarkers
Source: PLoS One. 2025 Jun 30;20(6):e0327594. doi: 10.1371/journal.pone.0327594 (PMC12208496; doi:10.1371/journal.pone.0327594)
Supplement: S2 Appendix — (DOCX) [file pone.0327594.s002.docx]

Alfaro AC, Thomas F, Sergent L, Duxbury M. Identification of trophic interactions within an estuarine food web (northern New Zealand) using fatty acid biomarkers and stable isotopes. Estuar Coast Shelf Sci. 2006;70(1–2):271–86. doi: [10.1016/j.ecss.2006.06.017](https://doi.org/10.1016/j.ecss.2006.06.017)

Budge SM, Parrish CC. Lipid biogeochemistry of plankton, settling matter and sediments in Trinity Bay, Newfoundland. II. Fatty acids. Org Geochem. 1998;29(5–7):1547–59. doi: [10.1016/S0146-6380(98)00177-6](https://doi.org/10.1016/S0146-6380(98)00177-6)

Budge SM, Parrish CC, Mckenzie CH. Fatty acid composition of phytoplankton, settling particulate matter and sediments at a sheltered bivalve aquaculture site. Mar Chem. 2001;76(4):285–303. doi: [10.1016/S0304-4203(01)00068-8](https://doi.org/10.1016/S0304-4203(01)00068-8)

Claustre H, Marty JC, Cassiani L, Dagaut J. Fatty acid dynamics in phytoplankton and microzooplankton communities during a spring bloom in the coastal Ligurian Sea: ecological implications. Marine Microbial Food Webs. 1988;3(2):51–66.

Dalsgaard J, St John M, Kattner G, Müller-Navarra D, Hagen W. Fatty acid trophic markers in the pelagic marine environment. Adv Mar Biol. 2003;46:225–340. [doi: 10.1016/S0065-2881(03)46005-7](https://doi.org/10.1016/S0065-2881(03)46005-7)

Harvey HR. Fatty acids and sterols as source markers of organic matter in sediments of the North Carolina continental slope. Deep Sea Res Part 2 Top Stud Oceanogr. 1994;41(4–6):783–96. doi: [10.1016/0031-9422(88)80432-1](https://doi.org/10.1016/0031-9422(88)80432-1)

Jónasdóttir SH. Fatty acid profiles and production in marine phytoplankton. Mar Drugs. 2019;17(3). doi: [10.3390/md17030151](https://doi.org/10.3390/md17030151)

Kharlamenko VI, Zhukova NV, Khotimchenko SV, Svetashev VI, Kamenev GM. Fatty acids as markers of food sources in a shallow-water hydrothermal ecosystem (Kraternaya Bight, Yankich Island, Kurile Islands). Mar Ecol Prog Ser. 1995;120:231–41. doi: [10.3354/meps120231](https://doi.org/10.3354/meps120231)

Khotimchenko SV, Vaskovsky VE, Titlyanova TV. Fatty acids of marine algae from the Pacific coast of North California. Bot Mar. 2002;45(1):17–22. doi: [10.1515/BOT.2002.003](https://doi.org/10.1515/BOT.2002.003)

Napolitano GE, Pollero RJ, Gayoso AM, Macdonald BA, Thompson RJ. Fatty acids as trophic markers of phytoplankton blooms in the Bahía Blanca estuary (Buenos Aires, Argentina) and in Trinity Bay (Newfoundland, Canada). Biochem Syst Ecol. 1997;25(8):739–55. doi: [10.1016/S0305-1978(97)00053-7](https://doi.org/10.1016/S0305-1978(97)00053-7)

Nichols PD, Jones GJ, De Leeuw JW, Johns RB. The fatty acid and sterol composition of two marine dinoflagellates. Phytochemistry. 1984;23(5):1043–7. doi: [10.1016/S0031-9422(00)82605-9](https://doi.org/10.1016/S0031-9422(00)82605-9)

Perry GJ, Volkman JK, Johns RB, Bavor HJ Jr. Fatty acids of bacterial origin in contemporary marine sediments. Geochim Cosmochim Acta. 1979;43(11):1715–25. doi: [10.1016/0016-7037(79)90020-6](https://doi.org/10.1016/0016-7037(79)90020-6)

Sargent JR, Parkes RJ, Muller-Harvey L, Henderson RJ. Lipid biomarkers in marine ecology. In: Sleigh MA, editor. Microbes in the Sea. Wiley and Sons; 1987. pp. 119–38.

Viso A-C, Marty J-C. Fatty acids from 28 marine microalgae. Phytochemistry. 1993;34(6):1521–33. doi: [10.1016/S0031-9422(00)90839-2](https://doi.org/10.1016/S0031-9422(00)90839-2)

Volkman JK, Johns RB, Gillan FT, Perry GJ, Bavor HJ Jr. Microbial lipids of an intertidal sediment—I. Fatty acids and hydrocarbons. Geochim Cosmochim Acta. 1980;44(8):1133–43. doi: [10.1016/0016-7037(80)90067-8](https://doi.org/10.1016/0016-7037(80)90067-8)

Yang D, Nam S, Hwang S-J, An K-G, Park Y-S, Shin K-H, et al. Fatty acid biomarkers to verify cyanobacteria feeding abilities of herbivorous consumers. J Freshw Ecol. 2016;31(1):77–91. doi: [10.1080/02705060.2015.1025304](https://doi.org/10.1080/02705060.2015.1025304)
