# Supplementary figures and images for: Characterizing the trophic ecology of herbivorous coral reef fishes using stable isotope and fatty acid biomarkers
Source: PLoS One. 2025 Jun 30;20(6):e0327594. doi: 10.1371/journal.pone.0327594 (PMC12208496; doi:10.1371/journal.pone.0327594)

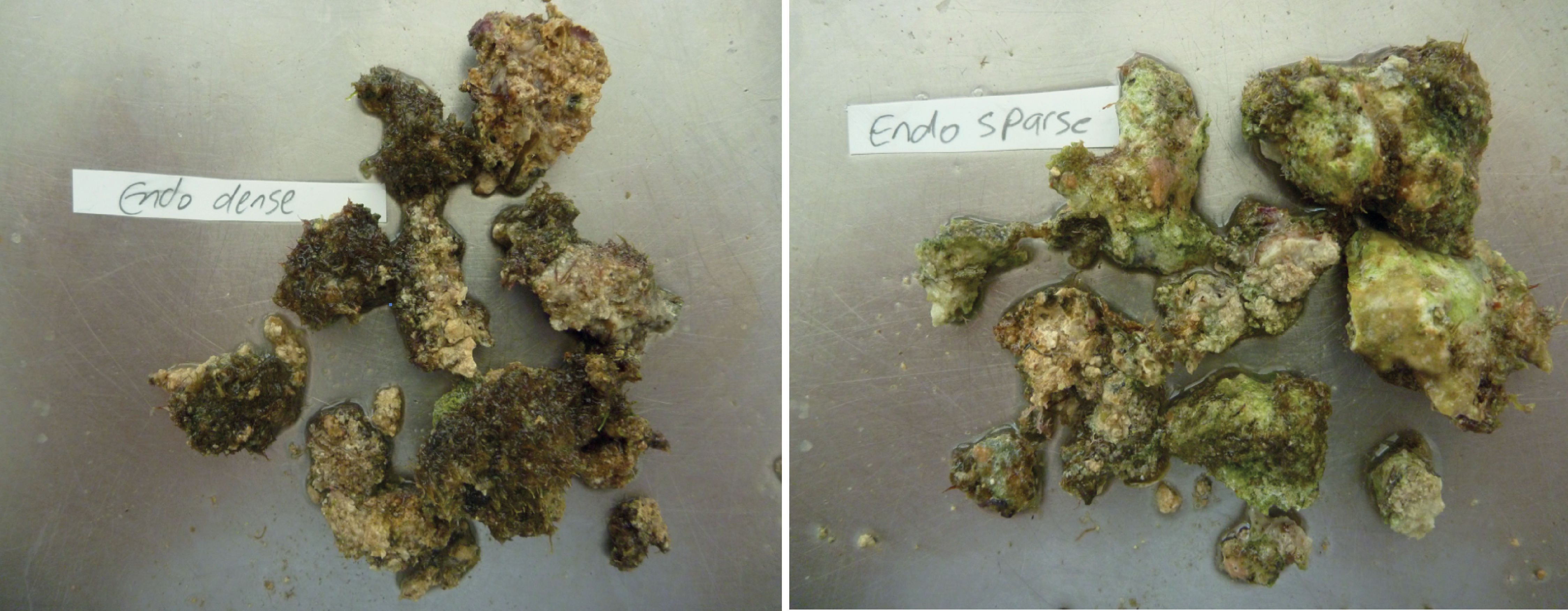

Supplement: S1 Fig — Left: endoliths and dense turf. Right: endoliths and sparse turf. (TIF) [file pone.0327594.s005.tif]

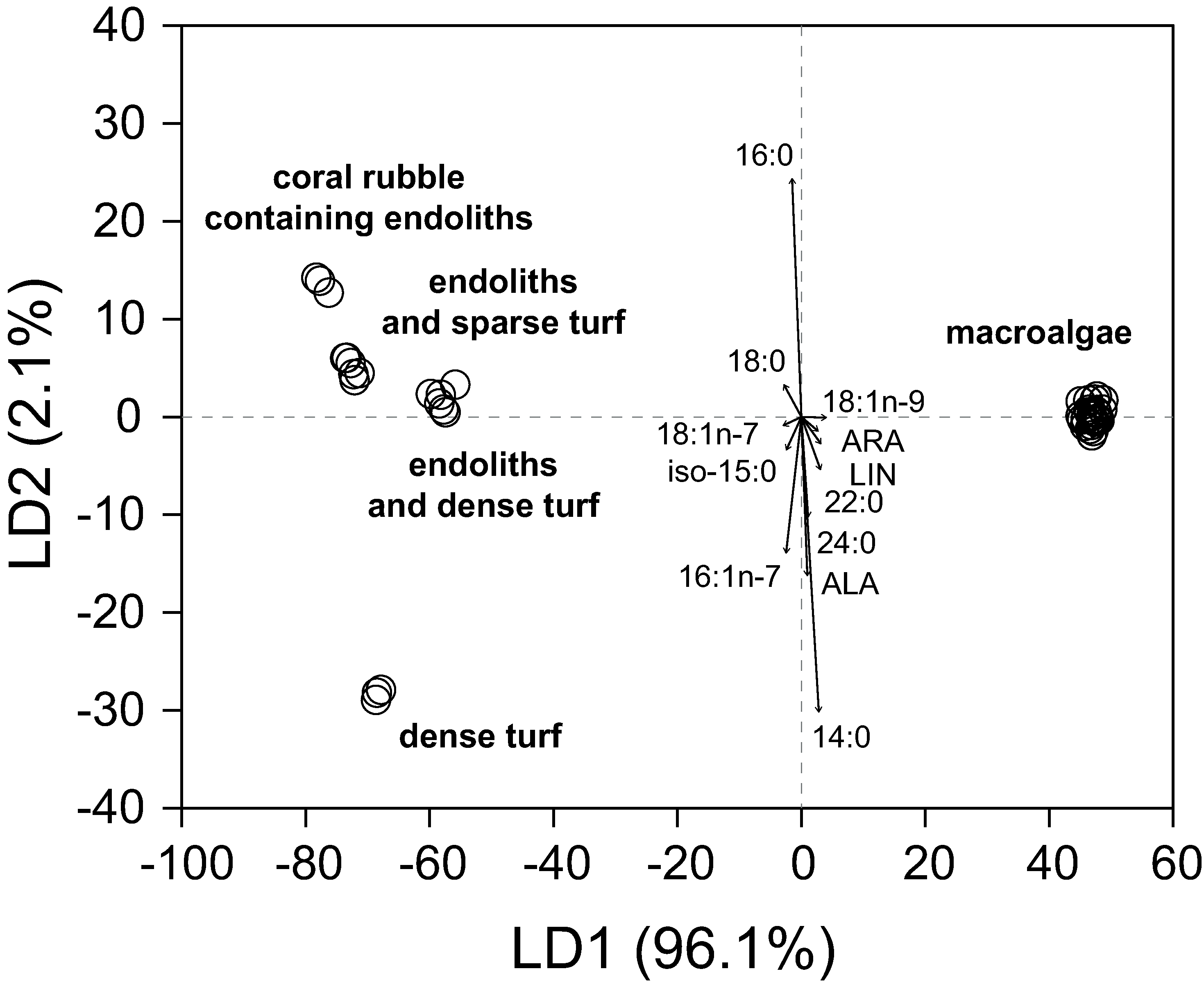

Supplement: S2 Fig — LD1 explains 96.1% of the variation and LD2 explains 2.1%. Sources are grouped in three clusters (dense turf, macroalgae and endolithic categories) according to their FA composition (PERMANOVA, F2,41 = 5.75, p < 0.001). Vectors indicate those individual FA contributing most to the overall variance among groups (SIMPER). For FA abbreviations, refer to S3 Table. (TIF) [file pone.0327594.s006.tif]
