# Supplementary material for: Characterizing the trophic ecology of herbivorous coral reef fishes using stable isotope and fatty acid biomarkers
Source: PLoS One. 2025 Jun 30;20(6):e0327594. doi: 10.1371/journal.pone.0327594 (PMC12208496; doi:10.1371/journal.pone.0327594)
Supplement: S1 Table — IP (initial phase) is equivalent to female (F) and TP (terminal phase) is equivalent to male (M) except in Chlorurus microrhinos and Scarus niger, where both sexes are TP. (DOCX) [file pone.0327594.s007.docx]

|  | **Species** | **n** | **SL (mm)** | **FL (mm)** | **TW (g)** | **Sex ratio (M/F)** | **Color phase** |
| --- | --- | --- | --- | --- | --- | --- | --- |
| Acanthuridae | *Acanthurus lineatus* | 7 | 180 – 201 | 210 – 231 | 290 – 370 | 0:1 | - |
|  | *A. nigrofuscus* | 4 | 120 – 144 | 140 – 166 | 73 – 124 | 0.2:1 | - |
|  | *Ctenochaetus striatus* | 6 | 151 – 185 | 180 – 220 | 173 – 292 | 1:0.3 | - |
|  | *Naso tonganus* | 9 | 240 – 470 | 275 – 508 | 392 – 2196 | 0.2:1 | - |
|  | *N. unicornis* | 11 | 226 – 425 | 256 – 475 | 348 – 2247 | 0.3:1 | - |
|  | *Zebrasoma velifer* | 7 | 150 – 194 | 180 – 230 | 154 – 282 | 1:0.3 | - |
| Kyphosidae | *Kyphosus cinerascens* | 7 | 260 – 300 | 292 – 339 | 636 – 1083 | 0.3:1 | - |
|  | *K. vaigiensis* | 7 | 225 – 296 | 258 – 333 | 377 – 816 | 0.1:1 | - |
| Pomacanthidae | *Pomacanthus sexstriatus* | 5 | 190 – 242 | 235 – 294 | 381 – 769 | 1:0.2 | - |
| Labridae (Scarinae) | *Chlorurus microrhinos* | 6 | 245 – 380 | 291 – 433 | 576 – 1880 | - | TP |
|  | *C. spilurus* | 6 | 209 – 245 | 241 – 286 | 326 – 509 | - | TP |
|  | *Scarus frenatus* | 6 | 252 – 290 | 300 – 350 | 622 – 923 | - | TP |
|  | *S. ghobban* | 4 | 233 – 330 | 295 – 385 | 517 – 1297 | - | IP |
|  | *S. niger* | 6 | 246 – 263 | 293 – 312 | 574 – 504 | - | TP |
|  | *S. rivulatus* | 6 | 249 – 260 | 298 – 307 | 602 – 745 | - | TP |
|  | *S. schlegeli* | 6 | 215 – 249 | 253 – 295 | 347 – 542 | - | TP |
|  | *S. spinus* | 1 | 185 | 217 | 236 | - | TP |
| Siganidae | *Siganus doliatus* | 7 | 160 – 190 | 183 – 219 | 146 – 265 | 0.4:1 | - |
