# Supplementary material for: Characterizing the trophic ecology of herbivorous coral reef fishes using stable isotope and fatty acid biomarkers
Source: PLoS One. 2025 Jun 30;20(6):e0327594. doi: 10.1371/journal.pone.0327594 (PMC12208496; doi:10.1371/journal.pone.0327594)
Supplement: S2 Table — Mean C:N ratio values (± sd) are also shown. P: Phaeophyceae, C: Chlorophyta, R: Rhodophyta. (DOCX) [file pone.0327594.s008.docx]

|  | **Sample type** | **Species** | **n** | **δ^13^C** | **δ^15^N** | **C:N** |
| --- | --- | --- | --- | --- | --- | --- |
| Organic matter sources | Dense turf |  | 9 | -11.1 ± 1.1 | 1.2 ± 0.2 | 6.9 ± 0.8 |
|  | Endoliths and dense turf | | 6 | -10.8 ± 1.1 | 2.5 ± 0.4 | 7.1 ± 1.0 |
|  | Endoliths and sparse turf | | 6 | -10.8 ± 2.0 | 2.8 ± 0.9 | 7.7 ± 1.4 |
|  | Coral rubble containing endoliths | | 3 | -13.7 ± 0.8 | 2.5 ± 0.5 | 7.0 ± 0.5 |
|  | Macroalgae | Total | 68 | -13.4 ± 5.5 | 2.3 ± 0.8 | 13.6 ± 11.7 |
|  |  | *Padina boergesenii^P^* | 7 | -9.7 ± 0.8 | 1.9 ± 0.3 | 13.5 ± 0.3 |
|  |  | *Turbinaria conoides* (thallus)*^P^* | 7 | -9.2 ± 1.7 | 2.6 ± 0.5 | 48.0 ± 16.0 |
|  |  | *T. conoides* (reproductive tissue)*^P^* | 7 | -11.1 ± 1.1 | 2.5 ± 0.2 | 24.2 ± 3.8 |
|  |  | *Dictyosphaeria versluysii^C^* | 7 | -2.5 ± 0.8 | 0.3 ± 0.5 | 15.6 ± 1.5 |
|  |  | *Halimeda macrophysa^C^* | 7 | -16.5 ± 1.1 | 2.3 ± 0.2 | 7.7 ± 0.3 |
|  |  | *Galaxaura marginata^R^* | 7 | -15.9 ± 0.9 | 2.9 ± 0.2 | 9.8 ± 1.1 |
|  |  | *Ganonema farinosum^R^* | 7 | -15.6 ± 1.2 | 2.4 ± 0.2 | 7.8 ± 1.0 |
|  |  | *Halymenia durvillei^R^* | 7 | -21.2 ± 1.7 | 3.2 ± 0.2 | 10.1 ± 0.5 |
|  |  | *Ohelopapa flexilis^R^* | 3 | -13.6 ± 0.8 | 2.4 ± 0.1 | 12.0 ± 0.7 |
|  |  | *Polysiphonia* sp.*^R^* | 1 | -28.5 ± 0.0 | 1.9 ± 0.0 | 5.5 ± 0.0 |
|  |  | *Portieria hornemannii^R^* | 6 | -16.0 ± 2.3 | 2.5 ± 0.2 | 10.1 ± 0.9 |
|  |  | *Hypnea valentiae^R^* | 2 | -17.4 ± 0.8 | 2.1 ± 0.0 | 11.0 ± 0.5 |
| Fish | Acanthuridae | *Acanthurus lineatus* | 7 | -12.8 ± 0.4 | 6.7 ± 0.3 | 3.1 ± 0.0 |
|  |  | *A. nigrofuscus* | 4 | -13.1 ± 0.6 | 6.7 ± 0.3 | 3.0 ± 0.0 |
|  |  | *Ctenochaetus striatus* | 6 | -11.2 ± 0.2 | 6.6 ± 0.2 | 3.1 ± 0.1 |
|  |  | *Naso tonganus* | 9 | -13.6 ± 1.2 | 7.0 ± 0.5 | 3.1 ± 0.0 |
|  |  | *N. unicornis* | 11 | -10.1 ± 1.2 | 5.6 ± 0.5 | 3.1 ± 0.0 |
|  |  | *Zebrasoma velifer* | 7 | -14.3 ± 1.3 | 6.4 ± 0.3 | 3.1 ± 0.0 |
|  | Kyphosidae | *Kyphosus cinerascens* | 7 | -12.3 ± 0.8 | 6.9 ± 0.7 | 3.1 ± 0.0 |
|  |  | *K. vaigiensis* | 7 | -12.0 ± 1.4 | 7.9 ± 0.8 | 3.1 ± 0.0 |
|  | Pomacanthidae | *Pomacanthus sexstriatus* | 5 | -15.1 ± 1.1 | 9.4 ± 0.3 | 3.1 ± 0.0 |
|  | Labridae (Scarinae) | *Chlorurus microrhinos* | 6 | -12.0 ± 2.5 | 6.4 ± 0.7 | 3.1 ± 0.1 |
|  |  | *C. spilurus* | 6 | -10.4 ± 0.3 | 5.8 ± 0.4 | 3.1 ± 0.0 |
|  |  | *Scarus frenatus* | 6 | -11.1 ± 0.4 | 6.6 ± 0.4 | 3.1 ± 0.0 |
|  |  | *S. ghobban* | 4 | -15.6 ± 0.4 | 7.6 ± 0.2 | 3.1 ± 0.0 |
|  |  | *S. niger* | 6 | -12.2 ± 0.2 | 6.3 ± 0.2 | 3.1 ± 0.1 |
|  |  | *S. rivulatus* | 6 | -11.3 ± 0.4 | 5.9 ± 0.3 | 3.1 ± 0.0 |
|  |  | *S. schlegeli* | 6 | -11.6 ± 0.4 | 5.7 ± 0.2 | 3.0 ± 0.0 |
|  |  | *S. spinus* | 1 | -9.6 | 5.8 | 3.0 |
|  | Siganidae | *Siganus doliatus* | 7 | -14.7 ± 1.1 | 6.2 ± 0.6 | 3.1 ± 0.1 |
