# Supplementary material for: Characterizing the trophic ecology of herbivorous coral reef fishes using stable isotope and fatty acid biomarkers
Source: PLoS One. 2025 Jun 30;20(6):e0327594. doi: 10.1371/journal.pone.0327594 (PMC12208496; doi:10.1371/journal.pone.0327594)
Supplement: S3 Table — Background shading indicates the most abundant FA (i.e., 18, average proportions greater than 1% in at least one source type). Different letters indicate significant differences (Dunn’s post hoc after significant Kruskal-Wallis test, p < 0.05) between the main five organic matter sources or between the macroalgae groups. For those FA or FA trophic markers displaying significant differences across sources, the highest value is shown in boldface. Data are also shown for individual macroalgae groups. Phaeophyceae pooled data from Padina boergesenii and Turbinaria conoides, Chlorophyta pooled data from Dictyosphaeria versluysii and Halimeda macrophysa, and Rhodophyta pooled data from Galaxaura marginata, Ganonema farinosum and Portieria hornemannii. SFA: saturated FA, MUFA: monounsaturated FA, PUFA: polyunsaturated FA, LIN: linoleic acid, ALA: α-linolenic acid, ARA: arachidonic acid, EPA: eicosapentaenoic acid, DHA: docosahexaenoic acid, Σn-3 PUFA: sum of n-3 PUFA; Σn-6 PUFA: sum of n-6 PUFA, Σn-3/Σn-6: sum of n-3 PUFA/sum of n-6 PUFA. Branched FA (BrFA) are iso-14:0, iso-15:0 and iso-16:0. (DOCX) [file pone.0327594.s009.docx]

|  | **Organic matter sources** | | | | |  | Macroalgae groups | | |
| --- | --- | --- | --- | --- | --- | --- | --- | --- | --- |
| **Fatty acids (%)** | **Dense turf**  **n=3** | **Endoliths**  **and dense turf**  **n=6** | **Endoliths**  **and sparse turf**  **n=6** | **Coral rubble**  **containing endoliths**  **n=3** | **Macroalgae**  **n=26** |  | Phaeophyceae  n=13 | Chlorophyta  n=7 | Rhodophyta n=6 |
| 6:0 | 0.0 ± 0.0^b^ | 0.0 ± 0.0^ab^ | 0.3 ± 0.2^ab^ | **0.8 ± 0.2^a^** | 0.2 ± 0.1^ab^ |  | 0.1 ± 0.0 | 0.4 ± 0.3 | 0.0 ± 0.0 |
| 8:0 | 0.2 ± 0.0 | 0.1 ± 0.0 | 0.2 ± 0.0 | 0.7 ± 0.5 | 0.2 ± 0.0 |  | 0.1 ± 0.0 | 0.2 ± 0.0 | 0.2 ± 0.0 |
| 9:0 | 0.1 ± 0.0 | 0.1 ± 0.0 | 0.1 ± 0.0 | 0.1 ± 0.0 | 0.1 ± 0.0 |  | 0.1 ± 0.0^b^ | 0.2 ± 0.0^a^ | **0.2 ± 0.0^a^** |
| 10:0 | 0.2 ± 0.0^ab^ | 0.1 ± 0.0^ab^ | 0.1 ± 0.0^b^ | **1.2 ± 0.8^a^** | 0.1 ± 0.0^ab^ |  | 0.1 ± 0.0 | 0.1 ± 0.0 | 0.1 ± 0.0 |
| 11:0 | 0.0 ± 0.0^ab^ | 0.0 ± 0.0^ab^ | **0.1 ± 0.0^a^** | 0.1 ± 0.0^ab^ | 0.0 ± 0.0^b^ |  | 0.0 ± 0.0^b^ | 0.0 ± 0.0^a^ | **0.0 ± 0.0^a^** |
| 12:0 | 0.7 ± 0.1 | 0.4 ± 0.0 | 0.4 ± 0.0 | 0.8 ± 0.1 | 0.3 ± 0.0 |  | 0.3 ± 0.1 | 0.4 ± 0.1 | 0.4 ± 0.0 |
| 13:0 | 0.2 ± 0.0^ab^ | 0.4 ± 0.0^a^ | **1.1 ± 0.3^a^** | 0.3 ± 0.0^ab^ | 0.1 ± 0.0^b^ |  | 0.1 ± 0.0 | 0.1 ± 0.0 | 0.1 ± 0.0 |
| 14:0 | **21.1 ± 1.7^a^** | 10.5 ± 0.3^ab^ | 8.4 ± 0.7^b^ | 10.1 ± 0.6^ab^ | 14.6 ± 1.0^ab^ |  | **17.5 ± 0.5^a^** | 14.3 ± 2.4^a^ | 8.9 ± 1.4^b^ |
| 15:0 | 1.9 ± 0.2^ab^ | 2.3 ± 0.2^ab^ | **3.4 ± 0.5^a^** | 2.4 ± 0.2^ab^ | 1.9 ± 0.2^b^ |  | **2.5 ± 0.3^a^** | 1.0 ± 0.2^b^ | 1.7 ± 0.2^ab^ |
| 16:0 | 39.4 ± 6.3 | 58.8 ± 0.7 | 57.3 ± 2.2 | 54.2 ± 2.9 | 51.2 ± 2.8 |  | 42.3 ± 3.1^b^ | 56.7 ± 4.2^a^ | **64.1 ± 4.0^a^** |
| 17:0 | **2.4 ± 0.5^a^** | 1.6 ± 0.1^a^ | 1.5 ± 0.1^ab^ | 2.3 ± 0.1^a^ | 0.9 ± 0.2^b^ |  | 1.0 ± 0.1 | 0.8 ± 0.1 | 1.0 ± 0.2 |
| 18:0 | 8.5 ± 0.6 | 9.0 ± 0.3 | 9.2 ± 0.7 | 10.2 ± 0.9 | 6.9 ± 0.5 |  | 5.9 ± 0.4 | 9.0 ± 1.4 | 6.5 ± 0.7 |
| 20:0 | 0.4 ± 0.1 | 0.4 ± 0.0 | 0.5 ± 0.1 | 0.6 ± 0.0 | 0.8 ± 0.1 |  | **1.1 ± 0.2^a^** | 0.4 ± 0.1^b^ | 0.5 ± 0.1^b^ |
| 21:0 | 0.1 ± 0.0^ab^ | 0.1 ± 0.0^ab^ | 0.1 ± 0.0^a^ | **0.2 ± 0.0^a^** | 0.1 ± 0.0^b^ |  | 0.1 ± 0.0 | 0.1 ± 0.0 | 0.1 ± 0.0 |
| 22:0 | 0.4 ± 0.1^ab^ | 0.2 ± 0.0^b^ | 0.3 ± 0.0^ab^ | 0.3 ± 0.0^ab^ | **1.1 ± 0.2^a^** |  | 1.1 ± 0.2 | 0.7 ± 0.4 | 1.3 ± 0.4 |
| 24:0 | 0.6 ± 0.3 | 0.3 ± 0.0 | 0.3 ± 0.0 | 0.4 ± 0.1 | 1.7 ± 0.4 |  | 0.6 ± 0.1 | 2.3 ± 0.9 | 3.4 ± 1.0 |
| iso-14:0 | 0.2 ± 0.0 | 0.2 ± 0.0 | 0.2 ± 0.0 | 0.2 ± 0.0 | 0.2 ± 0.0 |  | 0.1 ± 0.0 | 0.2 ± 0.1 | 0.3 ± 0.2 |
| iso-15:0 | **1.5 ± 0.2^ab^** | 1.3 ± 0.1^a^ | 1.4 ± 0.1^a^ | 1.3 ± 0.3^ab^ | 0.5 ± 0.1^b^ |  | 0.3 ± 0.1^b^ | 0.6 ± 0.1^ab^ | **0.9 ± 0.2^a^** |
| iso-16:0 | 0.4 ± 0.0^ab^ | **0.6 ± 0.0^a^** | 0.5 ± 0.1^a^ | 0.6 ± 0.1^a^ | 0.2 ± 0.0^b^ |  | 0.1 ± 0.0 | 0.2 ± 0.0 | 0.3 ± 0.1 |
| ΣSFA | 78.7 ± 3.2 | 86.5 ± 0.5 | 85.2 ± 0.7 | 86.7 ± 0.4 | 81.1 ± 1.9 |  | 73.5 ± 2.0^b^ | 87.8 ± 2.2^a^ | **89.9 ± 1.5^a^** |
| 16:1n-7 | **5.3 ± 0.3^a^** | 3.2 ± 0.2^ab^ | 2.9 ± 0.3^ab^ | 2.3 ± 0.2^ab^ | 2.1 ± 0.2^b^ |  | **2.9 ± 0.3^a^** | 1.5 ± 0.4^b^ | 1.2 ± 0.2^b^ |
| 16:1n-7t | 0.6 ± 0.1 | 0.4 ± 0.0 | 0.5 ± 0.1 | 0.4 ± 0.0 | 0.9 ± 0.2 |  | 0.9 ± 0.2 | 1.1 ± 0.4 | 0.7 ± 0.2 |
| 17:1n-7 | 0.3 ± 0.1^ab^ | 0.4 ± 0.1^ab^ | 0.3 ± 0.0^ab^ | **0.5 ± 0.0^a^** | 0.2 ± 0.0^b^ |  | **0.3 ± 0.0^a^** | 0.2 ± 0.1^b^ | 0.1 ± 0.0^b^ |
| 18:1n-7 | 2.3 ± 0.3^ab^ | **2.4 ± 0.1^a^** | 2.3 ± 0.1^ab^ | 2.2 ± 0.1^ab^ | 1.4 ± 0.2^b^ |  | 0.8 ± 0.2^b^ | **2.4 ± 0.5^a^** | 1.3 ± 0.2^ab^ |
| 18:1n-9 | 2.4 ± 0.4^ab^ | 1.9 ± 0.1^b^ | 2.4 ± 0.2^ab^ | 2.3 ± 0.1^ab^ | **4.9 ± 0.5^a^** |  | **6.7 ± 0.2^a^** | 2.3 ± 0.8^b^ | 4.2 ± 0.8^b^ |
| 20:1n-9 | 1.1 ± 0.3 | 0.3 ± 0.0 | 0.3 ± 0.0 | 0.3 ± 0.0 | 0.7 ± 0.1 |  | **1.1 ± 0.1^a^** | 0.5 ± 0.2^b^ | 0.2 ± 0.0^b^ |
| 22:1n-9t | 0.4 ± 0.1 | 0.3 ± 0.0 | 0.4 ± 0.0 | 0.3 ± 0.0 | 0.5 ± 0.1 |  | **0.8 ± 0.1^a^** | 0.2 ± 0.0^b^ | 0.1 ± 0.0^b^ |
| 24:1n-9 | 0.1 ± 0.0 | 0.0 ± 0.0 | 0.1 ± 0.0 | 0.1 ± 0.0 | 0.1 ± 0.0 |  | 0.0 ± 0.0 | 0.1 ± 0.0 | 0.1 ± 0.0 |
| ΣMUFA | 12.5 ± 1.1 | 9.0 ± 0.3 | 9.2 ± 0.3 | 8.4 ± 0.3 | 10.8 ± 0.8 |  | **13.4 ± 0.7^a^** | 8.2 ± 1.2^b^ | 7.9 ± 1.3^b^ |
| 18:2n-6 (LIN) | 2.0 ± 0.6 | 1.6 ± 0.1 | 2.0 ± 0.1 | 2.2 ± 0.1 | 1.9 ± 0.3 |  | **3.0 ± 0.3^a^** | 1.2 ± 0.4^b^ | 0.3 ± 0.1^b^ |
| 18:2n-6t | **0.5 ± 0.1^ab^** | 0.1 ± 0.0^c^ | 0.1 ± 0.0^cd^ | 0.0 ± 0.0^cd^ | 0.2 ± 0.0^bd^ |  | 0.2 ± 0.0 | 0.2 ± 0.1 | 0.2 ± 0.1 |
| 18:3n-3 (ALA) | 2.8 ± 0.8 | 0.8 ± 0.1 | 0.8 ± 0.1 | 0.6 ± 0.1 | 1.9 ± 0.4 |  | **3.2 ± 0.4^a^** | 1.0 ± 0.6^b^ | 0.2 ± 0.1^b^ |
| 18:3n-6 | 0.4 ± 0.2 | 0.2 ± 0.0 | 0.4 ± 0.0 | 0.2 ± 0.0 | 0.2 ± 0.0 |  | **0.3 ± 0.1^a^** | 0.1 ± 0.1^ab^ | 0.1 ± 0.0^b^ |
| 20:2n-6 | 0.2 ± 0.0 | 0.1 ± 0.0 | 0.2 ± 0.0 | 0.2 ± 0.0 | 0.2 ± 0.0 |  | **0.3 ± 0.1^a^** | 0.1 ± 0.0^b^ | 0.1 ± 0.0^b^ |
| 20:3n-6 | 0.1 ± 0.0 | 0.1 ± 0.0 | 0.1 ± 0.0 | 0.1 ± 0.0 | 0.3 ± 0.1 |  | **0.5 ± 0.1^a^** | 0.1 ± 0.0^b^ | 0.1 ± 0.0^b^ |
| 20:4n-6 (ARA) | 1.1 ± 0.3 | 0.9 ± 0.1 | 1.2 ± 0.1 | 0.9 ± 0.1 | 1.5 ± 0.3 |  | **2.8 ± 0.4^a^** | 0.3 ± 0.1^b^ | 0.2 ± 0.1^b^ |
| 20:5n-3 (EPA) | 0.8 ± 0.2 | 0.3 ± 0.0 | 0.6 ± 0.1 | 0.3 ± 0.0 | 0.4 ± 0.1 |  | 0.5 ± 0.1 | 0.3 ± 0.1 | 0.5 ± 0.1 |
| 22:2n-6 | 0.1 ± 0.0 | 0.1 ± 0.0 | 0.1 ± 0.0 | 0.0 ± 0.0 | 0.2 ± 0.0 |  | **0.2 ± 0.0^a^** | 0.0 ± 0.0^b^ | 0.0 ± 0.0^b^ |
| 22:4n-6 | 0.1 ± 0.0 | 0.1 ± 0.0 | 0.1 ± 0.0 | 0.1 ± 0.0 | 0.0 ± 0.0 |  | 0.0 ± 0.0 | 0.0 ± 0.0 | 0.0 ± 0.0 |
| 22:5n-3 | 0.0 ± 0.0 | 0.0 ± 0.0 | 0.0 ± 0.0 | 0.0 ± 0.0 | 0.0 ± 0.0 |  | 0.0 ± 0.0 | 0.0 ± 0.0 | 0.0 ± 0.0 |
| 22:5n-6 | 0.0 ± 0.0 | 0.0 ± 0.0 | 0.0 ± 0.0 | 0.0 ± 0.0 | 0.0 ± 0.0 |  | 0.0 ± 0.0 | 0.0 ± 0.0 | 0.0 ± 0.0 |
| 22:6n-3 (DHA) | 0.1 ± 0.0 | 0.0 ± 0.0 | 0.0 ± 0.0 | 0.0 ± 0.0 | 0.0 ± 0.0 |  | 0.0 ± 0.0 | 0.0 ± 0.0 | 0.0 ± 0.0 |
| Other PUFA | 0.6 ± 0.1^ab^ | 0.2 ± 0.0^b^ | 0.2 ± 1.0^b^ | 0.2 ± 0.0^b^ | **1.3 ± 0.2^a^** |  | **2.0 ± 0.4^a^** | 0.5 ± 0.1^b^ | 0.5 ± 0.1^b^ |
| ΣPUFA | 8.8 ± 2.1 | 4.5 ± 0.4 | 5.5 ± 0.5 | 5.0 ± 0.2 | 8.1 ± 1.3 |  | **13.0 ± 1.6^a^** | 4.0 ± 1.0^b^ | 2.2 ± 0.3^b^ |
| Σn-3 PUFA | 3.8 ± 1.0 | 1.2 ± 0.1 | 1.5 ± 0.2 | 0.9 ± 0.2 | 2.4 ± 0.4 |  | **3.7 ± 0.5^a^** | 1.4 ± 0.7^b^ | 0.8 ± 0.2^b^ |
| Σn-6 PUFA | 4.4 ± 1.0 | 3.1 ± 0.3 | 3.8 ± 0.2 | 3.8 ± 0.1 | 4.4 ± 0.7 |  | **7.3 ± 0.8^a^** | 2.0 ± 0.5^b^ | 0.9 ± 0.1^b^ |
| Σn-3/Σn-6 | **0.9 ± 0.0^a^** | 0.4 ± 0.0^ab^ | 0.4 ± 0.0^ab^ | 0.2 ± 0.0^b^ | 0.6 ± 0.1^ab^ |  | 0.5 ± 0.0 | 0.7 ± 0.2 | 0.9 ± 0.1 |
| 16:1n-7/16:0 | 0.1 ± 0.0 | 0.0 ± 0.0 | 0.0 ± 0.0 | 0.0 ± 0.0 | 0.1 ± 0.0 |  | **0.1 ± 0.0^a^** | 0.0 ± 0.0^b^ | 0.0 ± 0.0^b^ |
| Σ18:0 | 19.0 ± 2.6 | 15.9 ± 0.6 | 17.0 ± 0.8 | 17.7 ± 1.0 | 17.3 ± 0.9 |  | **20.0 ± 1.0^a^** | 16.2 ± 1.4^b^ | 12.8 ± 1.6^b^ |
| Σ16:0/Σ18:0 | 2.6 ± 0.7 | 4.0 ± 0.2 | 3.7 ± 0.3 | 3.3 ± 0.3 | 0.1 ± 0.0 |  | 2.5 ± 0.3^b^ | 4.1 ± 0.9^a^ | **5.9 ± 1.3^a^** |
| DHA/EPA | 0.1 ± 0.0 | 0.1 ± 0.0 | 0.1 ± 0.0 | 0.2 ± 0.0 | 0.1 ± 0.0 |  | 0.1 ± 0.0 | 0.2 ± 0.0 | 0.0 ± 0.0 |
| BrFA | 2.1 ± 0.2^ab^ | 2.0 ± 0.1^a^ | **2.1 ± 0.1^a^** | 2.1 ± 0.4^ab^ | 0.9^b^ ± 0.1 |  | 0.6 ± 0.1^b^ | 1.0 ± 0.2^a^ | **1.5 ± 0.4^a^** |
| 18:1n-7/18:1n-9 | 1.0 ± 0.1^ab^ | **1.3 ± 0.1^a^** | 1.0 ± 0.1^ab^ | 0.9 ± 0.1^ab^ | 0.3 ± 0.3^b^ |  | 0.1 ± 0.0^b^ | **1.1 ± 0.7^a^** | 0.3 ± 0.1^b^ |
