# Supplementary material for: Characterizing the trophic ecology of herbivorous coral reef fishes using stable isotope and fatty acid biomarkers
Source: PLoS One. 2025 Jun 30;20(6):e0327594. doi: 10.1371/journal.pone.0327594 (PMC12208496; doi:10.1371/journal.pone.0327594)
Supplement: S4 Table — Background shading indicates the most abundant FA (i.e., 21, average proportions greater than 1% in at least one species). Different letters indicate significant differences (Dunn’s post hoc after significant Kruskal-Wallis test, p < 0.05) between species. For those FA or FA trophic markers displaying significant differences across species, the highest values are shown in boldface. For FA abbreviations, refer to S3 Table. (DOCX) [file pone.0327594.s010.docx]

|  | **Acanthuridae** | | | | | | **Kyphosidae** | | **Pomacanthidae** | **Labridae (Scarinae)** | | | | | | | | **Siganidae** |
| --- | --- | --- | --- | --- | --- | --- | --- | --- | --- | --- | --- | --- | --- | --- | --- | --- | --- | --- |
| **Fatty acids (%)** | ***A. lineatus***  **n=7** | ***A. nigrofuscus***  **n=4** | ***C. striatus***  **n=6** | ***N. tonganus***  **n=9** | ***N. unicornis***  **n=11** | ***Z. velifer***  **n=7** | ***K. cinerascens***  **n=7** | ***K. vaigiensis***  **n=7** | ***P. sexstriatus***  **n=5** | ***C. microrhinos***  **n=6** | ***C. spilurus***  **n=6** | ***S. frenatus***  **n=6** | ***S. ghobban***  **n=4** | ***S. niger***  **n=6** | ***S. rivulatus***  **n=6** | ***S. schlegeli***  **n=6** | ***S. spinus***  **n=1** | ***S. doliatus***  **n=7** |
| 6:0 | 0.0 ± 0.0 | 0.1 ± 0.0 | 0.1 ± 0.0 | 0.1 ± 0.0 | 0.0 ± 0.0 | 0.0 ± 0.0 | 0.0 ± 0.0 | 0.1 ± 0.0 | 0.1 ± 0.0 | 0.1 ± 0.0 | 0.1 ± 0.0 | 0.0 ± 0.0 | 0.0 ± 0.0 | 0.1 ± 0.0 | 0.1 ± 0.0 | 0.1 ± 0.0 | 0.0 | 0.1 ± 0.0 |
| 8:0 | 0.0 ± 0.0 | 0.0 ± 0.0 | 0.1 ± 0.0 | 0.0 ± 0.0 | 0.0 ± 0.0 | 0.0 ± 0.0 | 0.0 ± 0.0 | 0.0 ± 0.0 | 0.0 ± 0.0 | 0.0 ± 0.0 | 0.0 ± 0.0 | 0.0 ± 0.0 | 0.0 ± 0.0 | 0.0 ± 0.0 | 0.0 ± 0.0 | 0.0 ± 0.0 | 0.0 | 0.0 ± 0.0 |
| 9:0 | 0.0 ± 0.0 | 0.0 ± 0.0 | 0.1 ± 0.1 | 0.0 ± 0.0 | 0.0 ± 0.0 | 0.0 ± 0.0 | 0.1 ± 0.1 | 0.0 ± 0.0 | 0.0 ± 0.0 | 0.0 ± 0.0 | 0.0 ± 0.0 | 0.0 ± 0.0 | 0.0 ± 0.0 | 0.0 ± 0.0 | 0.0 ± 0.0 | 0.0 ± 0.0 | 0.0 | 0.0 ± 0.0 |
| 10:0 | **0.1 ± 0.0^a^** | 0.0 ± 0.0^ab^ | 0.0 ± 0.0^ab^ | 0.0 ± 0.0^ab^ | 0.0 ± 0.0^b^ | 0.0 ± 0.0^b^ | 0.0 ± 0.0^ab^ | 0.0 ± 0.0^ab^ | 0.0 ± 0.0^ab^ | 0.0 ± 0.0^ab^ | 0.0 ± 0.0^ab^ | 0.0 ± 0.0^ab^ | 0.0 ± 0.0^ab^ | 0.0 ± 0.0^ab^ | 0.0 ± 0.0^ab^ | 0.0 ± 0.0^ab^ | 0.0 | 0.1 ± 0.0^ab^ |
| 11:0 | 0.0 ± 0.0^ab^ | 0.0 ± 0.0^ab^ | 0.0 ± 0.0^ab^ | 0.0 ± 0.0^ab^ | 0.0 ± 0.0^ab^ | 0.0 ± 0.0^b^ | 0.0 ± 0.0^ab^ | 0.0 ± 0.0^ab^ | 0.0 ± 0.0^ab^ | 0.0 ± 0.0^ab^ | 0.0 ± 0.0^ab^ | 0.0 ± 0.0^ab^ | 0.0 ± 0.0^ab^ | 0.0 ± 0.0^ab^ | 0.0 ± 0.0^ab^ | 0.0 ± 0.0^ab^ | 0.0 | **0.0 ± 0.0^a^** |
| 12:0 | 0.3 ± 0.0^ab^ | 0.1 ± 0.0^abcd^ | 0.2 ± 0.0^abcd^ | 0.1 ± 0.0^abcd^ | 0.1 ± 0.0^abcd^ | 0.1 ± 0.0^abcd^ | 0.3 ± 0.1^abd^ | 0.1 ± 0.0^abcd^ | 0.1 ± 0.0^abcd^ | 0.0 ± 0.0^c^ | 0.1 ± 0.0^cd^ | 0.1 ± 0.0^c^ | 0.1 ± 0.0^abcd^ | 0.0 ± 0.0^c^ | 0.1 ± 0.0^bcd^ | 0.0 ± 0.0^cd^ | 0.1 | **1.9 ± 0.7^a^** |
| 13:0 | **0.1 ± 0.0^a^** | 0.0 ± 0.0^abcd^ | 0.1 ± 0.0^ad^ | 0.0 ± 0.0^abcd^ | 0.0. ± 0.0^abcd^ | 0.0 ± 0.0^abcd^ | 0.1 ± 0.0^a^ | 0.0 ± 0.0^abcd^ | 0.1 ± 0.0^a^ | 0.0 ± 0.0^abcd^ | 0.0 ± 0.0^bc^ | 0.0 ± 0.0^bcd^ | 0.0 ± 0.0^abcd^ | 0.0 ± 0.0^b^ | 0.0 ± 0.0^abcd^ | 0.0 ± 0.0^bc^ | 0.0 | 0.1 ± 0.0^acd^ |
| 14:0 | 14.2 ± 1.0^a^ | 6.8 ± 1.2^abc^ | **15.4 ± 2.8^ac^** | 5.3 ± 1.3^abc^ | 8.4 ± 0.9^ac^ | 7.3 ± 1.4^abc^ | 14.6 ± 1.6^a^ | 6.1 ± 1.9^abc^ | 7.6 ± 0.5^abc^ | 1.2 ± 0.1^b^ | 1.3 ± 0.1^b^ | 2.0 ± 0.3^bc^ | 2.1 ± 0.5^abc^ | 1.3 ± 0.1^b^ | 2.3 ± 0.4^bc^ | 1.9 ± 0.1^bc^ | 1.1 | 4.5 ± 0.7^abc^ |
| 15:0 | 2.8 ± 0.3^ab^ | 1.8 ± 0.3^abc^ | 3.0 ± 0.9^abc^ | 1.3 ± 0.2^abc^ | 1.9 ± 0.2^abc^ | 2.0 ± 0.2^abc^ | **4.1 ± 0.7^a^** | 1.3 ± 0.2^abc^ | 3.4 ± 0.5^ab^ | 1.2 ± 0.1^abc^ | 1.0 ± 0.1^bc^ | 1.0 ± 0.1^c^ | 1.5 ± 0.1^abc^ | 0.8 ± 0.1^c^ | 0.9 ± 0.2^c^ | 0.8 ± 0.1^c^ | 0.9 | 1.3 ± 0.1^abc^ |
| 16:0 | 36.4 ± 1.8 | 41.2 ± 1.9 | 37.1 ± 3.0 | 45.2 ± 1.5 | 40.4 ± 2.2 | 43.2 ± 2.0 | 33.6 ± 2.9 | 45.9 ± 3.0 | 36.2 ± 1.7 | 38.9 ± 1.3 | 40.4 ± 1.5 | 43.8 ± 1.0 | 42.5 ± 1.7 | 44.7 ± 1.3 | 42.5 ± 1.7 | 45.3 ± 1.1 | 46.0 | 45.8 ± 2.3 |
| 17:0 | 2.9 ± 0.2^ab^ | 2.3 ± 0.2^abc^ | 2.8 ± 0.8^abc^ | 2.2 ± 0.2^abc^ | 1.2 ± 0.2^c^ | 2.1 ± 0.1^abc^ | 3.2 ± 0.5^ab^ | 1.2 ± 0.1^c^ | **5.1 ± 0.6^a^** | 2.3 ± 0.2^abc^ | 2.5 ± 0.3^abc^ | 2.3 ± 0.1^abc^ | 3.5 ± 0.3^ab^ | 2.3 ± 0.1^abc^ | 2.5 ± 0.2^abc^ | 2.0 ± 0.1^abc^ | 2.6 | 1.7 ± 0.1^bc^ |
| 18:0 | 13.4 ± 0.5^d^ | 16.9 ± 1.2^abcd^ | 15.4 ± 1.0^bcd^ | 18.5 ± 0.9^abcd^ | 14.9 ± 0.4^cd^ | 15.7 ± 0.8^bcd^ | 14.1 ± 0.4^cd^ | 17.5 ± 1.7^abcd^ | 18.9 ± 0.2^abcd^ | **24.9 ± 0.6^a^** | 23.5 ± 0.9^a^ | 20.7 ± 0.3^abcd^ | 22.0 ± 0.7^abc^ | 21.8 ± 0.3^ab^ | 21.1 ± 0.3^abc^ | 20.8 ± 0.4^abc^ | 21.8 | 16.7 ± 0.8^abcd^ |
| 20:0 | 0.4 ± 0.0^abc^ | 0.3 ± 0.0^abc^ | 0.5 ± 0.2^abc^ | 0.2 ± 0.0^bc^ | 0.4 ± 0.1^abc^ | 0.3 ± 0.0^abc^ | **0.8 ± 0.2^a^** | 0.4 ± 0.1^abc^ | 0.5 ± 0.0^ab^ | 0.2 ± 0.0^abc^ | 0.3 ± 0.0^abc^ | 0.2 ± 0.0^bc^ | 0.2 ± 0.0^abc^ | 0.2 ± 0.0^c^ | 0.2 ± 0.0^bc^ | 0.2 ± 0.0^bc^ | 0.1 | 0.2 ± 0.0^abc^ |
| 21:0 | 0.1 ± 0.0^ab^ | 0.1 ± 0.0^ab^ | 0.1 ± 0.1^abc^ | 0.1 ± 0.0^c^ | 0.1 ± 0.0^c^ | 0.1 ± 0.0^abc^ | 0.2 ± 0.0^ab^ | 0.1 ± 0.0^abc^ | **0.2 ± 0.0^a^** | 0.1 ± 0.0^abc^ | 0.1 ± 0.0^abc^ | 0.1 ± 0.0^abc^ | 0.1 ± 0.0^abc^ | 0.1 ± 0.0^abc^ | 0.1 ± 0.0^abc^ | 0.1 ± 0.0^bc^ | 0.0 | 0.1 ± 0.0^abc^ |
| 22:0 | 0.1 ± 0.0^ab^ | 0.1 ± 0.0^ab^ | **0.3 ± 0.1^a^** | 0.1 ± 0.0^ab^ | 0.2 ± 0.0^ab^ | 0.1 ± 0.0^b^ | 0.3 ± 0.0^a^ | 0.3 ± 0.1^ab^ | 0.2 ± 0.0^a^ | 0.2 ± 0.0^a^ | 0.2 ± 0.0^ab^ | 0.2 ± 0.0^ab^ | 0.1 ± 0.0^ab^ | 0.2 ± 0.0^ab^ | 0.2 ± 0.0^ab^ | 0.2 ± 0.0^ab^ | 0.2 | 0.2 ± 0.0^ab^ |
| 24:0 | 0.2 ± 0.1 | 0.3 ± 0.1 | 0.3 ± 0.1 | 0.2 ± 0.1 | 0.1 ± 0.0 | 0.2 ± 0.1 | 0.1 ± 0.1 | 0.2 ± 0.0 | 0.2 ± 0.0 | 0.2 ± 0.0 | 0.3 ± 0.1 | 0.2 ± 0.0 | 0.1 ± 0.0 | 0.2 ± 0.0 | 0.2 ± 0.0 | 0.3 ± 0.1 | 0.2 | 0.1 ± 0.0 |
| iso-14:0 | 0.1 ± 0.0^a^ | 0.0 ± 0.0^abc^ | 0.1 ± 0.0^a^ | 0.0 ± 0.0^abc^ | 0.0 ± 0.0^abc^ | 0.0 ± 0.0^abc^ | 0.0 ± 0.0^ac^ | 0.0 ± 0.0^abc^ | **0.1 ± 0.0^a^** | 0.0 ± 0.0^b^ | 0.0 ± 0.0^bc^ | 0.0 ± 0.0^abc^ | 0.0 ± 0.0^abc^ | 0.0 ± 0.0^bc^ | 0.0 ± 0.0^abc^ | 0.0 ± 0.0^abc^ | 0.0 | 0.0 ± 0.0^abc^ |
| iso-15:0 | 0.4 ± 0.0^a^ | 0.2 ± 0.0^abc^ | 0.5 ± 0.1^a^ | 0.1 ± 0.0^bc^ | 0.0 ± 0.0^c^ | 0.1 ± 0.0^abc^ | 0.2 ± 0.0^ab^ | 0.1 ± 0.0^bc^ | **1.6 ± 0.4**^a^ | 0.1 ± 0.0^abc^ | 0.1 ± 0.0^abc^ | 0.1 ± 0.0^abc^ | 0.2 ± 0.0^ab^ | 0.1 ± 0.0^abc^ | 0.1 ± 0.0^abc^ | 0.1 ± 0.0^abc^ | 0.1 | 0.0 ± 0.0^c^ |
| iso-16:0 | 0.2 ± 0.0^a^ | 0.1 ± 0.0^abc^ | 0.4 ± 0.1^a^ | 0.1 ± 0.0^bc^ | 0.0 ± 0.0^c^ | 0.1 ± 0.0^abc^ | 0.1 ± 0.0^abc^ | 0.1 ± 0.0^bc^ | **0.5 ± 0.1^a^** | 0.1 ± 0.0^ab^ | 0.1 ± 0.0^abc^ | 0.1 ± 0.0^abc^ | 0.2 ± 0.0^a^ | 0.1 ± 0.0^abc^ | 0.1 ± 0.0^abc^ | 0.1 ± 0.0^abc^ | 0.1 | 0.0 ± 0.0^bc^ |
| ΣSFA | 71.7 ± 0.8 | 70.3 ± 1.3 | 76.6 ± 2.5 | 73.5 ± 1.0 | 67.9 ± 1.2 | 71.4 ± 1.5 | 71.9 ± 1.6 | 73.2 ± 3.0 | 74.8 ± 1.3 | 69.7 ± 1.2 | 70.1 ± 1.4 | 70.8 ± 0.7 | 72.9 ± 1.7 | 72.0 ± 0.9 | 70.4 ± 1.3 | 72.1 ± 0.8 | 73.3 | 73.0 ± 1.0 |
| 16:1n-7 | 4.1 ± 0.3^ab^ | 1.6 ± 0.2^abc^ | **5.9 ± 0.5^a^** | 1.6 ± 0.3^abc^ | 1.6 ± 0.2^abc^ | 2.6 ± 0.4^abc^ | 4.3 ± 0.3^ab^ | 1.6 ± 0.5^bc^ | 1.7 ± 0.2^abc^ | 0.9 ± 0.1^c^ | 0.8 ± 0.1^c^ | 1.3 ± 0.2^abc^ | 1.5 ± 0.3^abc^ | 0.9 ± 0.1^c^ | 2.1 ± 0.4^abc^ | 2.4 ± 0.1^abc^ | 0.6 | 1.1 ± 0.1^c^ |
| 16:1n-7t | 0.9 ± 0.3^ab^ | 0.2 ± 0.0^abc^ | 0.4 ± 0.2^abc^ | 0.2 ± 0.1^abc^ | 0.3 ± 0.1^abc^ | 0.6 ± 0.3^abc^ | **1.3 ± 0.2^a^** | 0.7 ± 0.3^abc^ | 0.3 ± 0.1^abc^ | 0.1 ± 0.0^c^ | 0.2 ± 0.1^abc^ | 0.3 ± 0.1^abc^ | 0.1 ± 0.0bc | 0.2 ± 0.1^bc^ | 0.3 ± 0.2^abc^ | 0.6 ± 0.3^abc^ | 0.1 | 0.4 ± 0.1^abc^ |
| 17:1n-7 | 0.2 ± 0.0^ab^ | 0.2 ± 0.0^ab^ | 0.2 ± 0.0^abc^ | 0.2 ± 0.0^abc^ | 0.2 ± 0.0^abc^ | **0.3 ± 0.0^a^** | 0.2 ± 0.0^abc^ | 0.1 ± 0.0^ac^ | 0.3 ± 0.0^ab^ | 0.1 ± 0.0^abc^ | 0.1 ± 0.0^c^ | 0.1 ± 0.0^c^ | 0.2 ± 0.0^abc^ | 0.1 ± 0.0^c^ | 0.1 ± 0.0^ac^ | 0.1 ± 0.0^abc^ | 0.1 | 0.2 ± 0.0^abc^ |
| 18:1n-7 | 1.5 ± 0.1^abcd^ | 1.3 ± 0.1^abcd^ | 2.3 ± 0.3^abc^ | 1.4 ± 0.1^abcd^ | 1.3 ± 0.2^bcd^ | 1.7 ± 0.2^abcd^ | 2.3 ± 0.4^abc^ | 0.7 ± 0.2^d^ | **2.8 ± 0.4^ab^** | 2.4 ± 0.1^ab^ | 2.6 ± 0.2^a^ | 2.2 ± 0.1^abc^ | 2.7 ± 0.1^ab^ | 1.8 ± 0.1^abcd^ | 2.2 ± 0.2^abc^ | 1.7 ± 0.1^abcd^ | 2.0 | 1.1 ± 0.1^cd^ |
| 18:1n-9 | 4.4 ± 0.1^abcde^ | 4.9 ± 0.2^abcde^ | 3.5 ± 0.2^de^ | 5.1 ± 0.1^abcd^ | **6.0 ± 0.1^a^** | 5.9 ± 0.2^ab^ | 5.4 ± 0.2^abc^ | 5.6 ± 0.1^abc^ | 3.3 ± 0.1^de^ | 4.6 ± 0.3^abcde^ | 3.9 ± 0.2^bcde^ | 3.5 ± 0.1^de^ | 4.1 ± 0.2^abcde^ | 3.8 ± 0.2^cde^ | 3.9 ± 0.1^bcde^ | 3.8 ± 0.2^cde^ | 4.2 | 4.3 ± 0.2^ab^ |
| 20:1n-9 | 0.8 ± 0.1^bc^ | 0.5 ± 0.0^abcd^ | 0.5 ± 0.1^abcd^ | 0.6 ± 0.1^abcd^ | 1.4 ± 0.2^ac^ | 1.2 ± 0.3^bc^ | **1.8 ± 0.3^ab^** | 0.6 ± 0.2^abcd^ | 0.3 ± 0.1^abcd^ | 0.2 ± 0.0^d^ | 0.3 ± 0.0^bd^ | 0.4 ± 0.0^abcd^ | 0.4 ± 0.1^abcd^ | 0.3 ± 0.0^bd^ | 0.5 ± 0.0^abcd^ | 0.4 ± 0.0^abcd^ | 0.3 | 0.2 ± 0.0^bc^ |
| 22:1n-9t | 0.9 ± 0.1^cd^ | 1.6 ± 0.1^abcd^ | 0.7 ± 0.3^cd^ | 1.7 ± 0.1^abcd^ | 1.6 ± 0.1^abcd^ | 1.3 ± 0.1^cd^ | 0.7 ± 0.1^c^ | 1.5 ± 0.3^abcd^ | 1.9 ± 0.2^abcd^ | 2.8 ± 0.1^ab^ | **3.0 ± 0.2^a^** | 2.7 ± 0.1^a^ | 2.0 ± 0.2^abcd^ | 2.8 ± 0.1^a^ | 2.3 ± 0.1^abd^ | 2.1 ± 0.1^abcd^ | 2.3 | 2.0 ± 0.1^abcd^ |
| 24:1n-9 | 0.1 ± 0.0^ab^ | 0.2 ± 0.0^ab^ | 0.1 ± 0.0^b^ | **0.2 ± 0.0^a^** | 0.2 ± 0.0^ab^ | 0.2 ± 0.0^ab^ | 0.2 ± 0.0^ab^ | 0.2 ± 0.0^ab^ | 0.2 ± 0.1^ab^ | 0.1 ± 0.0^ab^ | 0.1 ± 0.0^ab^ | 0.1 ± 0.0^ab^ | 0.1 ± 0.0^b^ | 0.1 ± 0.0^ab^ | 0.1 ± 0.0^b^ | 0.1 ± 0.0^b^ | 0.1 | 0.1 ± 0.0^ab^ |
| ΣMUFA | 12.9 ± 0.6^ab^ | 10.5 ± 0.4^abc^ | 13.7 ± 0.7^ab^ | 10.9 ± 0.5^abc^ | 12.6 ± 0.6^ab^ | 13.7 ± 1.2^ab^ | **16.2 ± 0.6^a^** | 11.1 ± 1.2^abc^ | 10.7 ± 0.5^abc^ | 11.2 ± 0.3^abc^ | 11.0 ± 0.5^abc^ | 10.6 ± 0.2^bc^ | 11.1 ± 0.4^abc^ | 10.0 ± 0.3^bc^ | 11.5 ± 0.4^abc^ | 11.3 ± 0.3^abc^ | 9.9 | 9.5 ± 0.3^c^ |
| 18:2n-6 (LIN) | 1.0 ± 0.1^bcd^ | 1.0 ± 0.1^bcd^ | 1.0 ± 0.2^cd^ | 0.8 ± 0.1^d^ | **2.3** **± 0.1^a^** | 0.9 ± 0.1^cd^ | 1.3 ± 0.2^abcd^ | 1.7 ± 0.2^abcd^ | 0.9 ± 0.1^d^ | 1.7 ± 0.0^abcd^ | 2.0 ± 0.0^ab^ | 1.6 ± 0.1^abcd^ | 1.7 ± 0.1^abcd^ | 1.2 ± 0.1^abcd^ | 1.5 ± 0.1^abcd^ | 1.2 ± 0.1^abcd^ | 1.6 | 1.3 ± 0.2^abcd^ |
| 18:2n-6t | 0.3 ± 0.0^a^ | 0.2 ± 0.0^acd^ | **0.3 ± 0.1^abcd^** | 0.1 ± 0.0^abcd^ | 0.1 ± 0.0^acd^ | 0.1 ± 0.0^abcd^ | 0.2 ± 0.0^ad^ | 0.2 ± 0.0^ad^ | 0.1 ± 0.0^abcd^ | 0.1 ± 0.0^abcd^ | 0.1 ± 0.0^abcd^ | 0.0 ± 0.0^bc^ | 0.0 ± 0.0^bc^ | 0.0 ± 0.0^bc^ | 0.0 ± 0.0^bc^ | 0.0 ± 0.0^b^ | 0.0 | 0.1 ± 0.0^bcd^ |
| 18:3n-3 (ALA) | **1.4** **± 0.1^a^** | 0.8 ± 0.1^ab^ | 0.7 ± 0.2^ab^ | 0.7 ± 0.2^ab^ | 0.9 ± 0.1^ab^ | 0.8 ± 0.2^ab^ | 0.8 ± 0.1^ab^ | 1.2 ± 0.4^ab^ | 0.7 ± 0.2^ab^ | 0.6 ± 0.1^ab^ | 0.7 ± 0.1^ab^ | 1.0 ± 0.1^ab^ | 0.9 ± 0.2^ab^ | 0.7 ± 0.0^ab^ | 1.2 ± 0.1^b^ | 0.9 ± 0.1^ab^ | 0.7 | 0.6 ± 0.1^b^ |
| 18:3n-6 | **0.9 ± 0.1^a^** | 0.6 ± 0.0^ab^ | 0.5 ± 0.2^ab^ | 0.2 ± 0.0^b^ | 0.9 ± 0.1^a^ | 0.2 ± 0.0^ab^ | 0.2 ± 0.0^b^ | 0.3 ± 0.1^ab^ | 0.2 ± 0.0^b^ | 0.3 ± 0.0^ab^ | 0.2 ± 0.0^ab^ | 0.2 ± 0.0^ab^ | 0.2 ± 0.0^ab^ | 0.2 ± 0.0^b^ | 0.2 ± 0.0^ab^ | 0.2 ± 0.0^b^ | 0.2 | 0.2 ± 0.0^ab^ |
| 20:2n-6 | 0.1 ± 0.0^c^ | 0.1 ± 0.0^abcd^ | 0.1 ± 0.0^cd^ | 0.1 ± 0.0^cd^ | 0.2 ± 0.0^ab^ | 0.1 ± 0.0^bcd^ | 0.2 ± 0.0^abcd^ | 0.2 ± 0.0^abcd^ | 0.1 ± 0.0^abcd^ | 0.3 ± 0.0^a^ | **0.3 ± 0.0^a^** | 0.2 ± 0.0^abd^ | 0.2 ± 0.0^abcd^ | 0.1 ± 0.0^abcd^ | 0.2 ± 0.0^ab^ | 0.1 ± 0.0^abcd^ | 0.1 | 0.1 ± 0.0^abcd^ |
| 20:3n-6 | 0.5 ± 0.0^abcd^ | 0.6 ± 0.1^abcd^ | 0.2 ± 0.0^c^ | 0.8 ± 0.1^ab^ | **1.3 ± 0.1^a^** | 0.6 ± 0.1^abcd^ | 0.7 ± 0.1^abcd^ | 0.7 ± 0.1^abd^ | 0.4 ± 0.1^abcd^ | 0.3 ± 0.0^bcd^ | 0.3 ± 0.0^bcd^ | 0.3 ± 0.0^bcd^ | 0.2 ± 0.0^bcd^ | 0.3 ± 0.0^bcd^ | 0.3 ± 0.0^bcd^ | 0.3 ± 0.0^cd^ | 0.3 | 0.7 ± 0.1^abd^ |
| 20:4n-6 (ARA) | 2.9 ± 0.2^cd^ | 5.3 ± 0.5^abcd^ | 2.2 ± 0.8^cd^ | 5.4 ± 0.4^abcd^ | 5.2 ± 0.4^abcd^ | 4.1 ± 0.4^bcd^ | 2.1 ± 0.4^cd^ | 4.5 ± 0.8^abcd^ | 6.0 ± 0.7^abcd^ | 9.0 ± 0.4^ab^ | **9.5** **± 0.5^a^** | 8.6 ± 0.3^a^ | 6.4 ± 0.8^abcd^ | 8.9 ± 0.3^a^ | 7.3 ± 0.5^abd^ | 6.5 ± 0.2^abcd^ | 7.3 | 6.2 ± 0.2^abcd^ |
| 20:5n-3 (EPA) | 3.2 ± 0.2^a^ | **3.9** **± 0.3^ab^** | 1.2 ± 0.4^b^ | 3.3 ± 0.3^ab^ | 2.8 ± 0.2^ab^ | 3.8 ± 0.4^a^ | 3.3 ± 0.5^ab^ | 2.8 ± 0.3^ab^ | 1.1 ± 0.2^b^ | 1.7 ± 0.4^ab^ | 1.6 ± 0.3^b^ | 1.9 ± 0.2^ab^ | 2.0 ± 0.4^ab^ | 2.2 ± 0.1^ab^ | 2.8 ± 0.2^ab^ | 3.2 ± 0.1^ab^ | 3.8 | 1.9 ± 0.1^ab^ |
| 22:2n-6 | 0.1 ± 0.0^abcd^ | 0.1 ± 0.0^abcd^ | 0.1 ± 0.0^bcd^ | 0.1 ± 0.0^abcd^ | **0.4 ± 0.0^a^** | 0.1 ± 0.0^abcd^ | 0.2 ± 0.0^ab^ | 0.3 ± 0.1^abd^ | 0.2 ± 0.0^abcd^ | 0.0 ± 0.0^c^ | 0.0 ± 0.0^c^ | 0.2 ± 0.0^bcd^ | 0.2 ± 0.0^bcd^ | 0.2 ± 0.0^cd^ | 0.2 ± 0.0^bcd^ | 0.2 ± 0.0^bcd^ | 0.2 | 0.2 ± 0.0^abd^ |
| 22:4n-6 | 0.7 ± 0.1^abcd^ | 0.8 ± 0.1^abcd^ | 0.2 ± 0.1^bc^ | 1.0 ± 0.1^ad^ | 1.1 ± 0.1^a^ | 0.9 ± 0.1^acd^ | 0.9 ± 0.1^abcd^ | 0.8 ± 0.2^abcd^ | **1.2** **± 0.1^a^** | 0.8 ± 0.1^abcd^ | 0.6 ± 0.0^abcd^ | 0.6 ± 0.0^abcd^ | 0.3 ± 0.0^bcd^ | 0.4 ± 0.0^bcd^ | 0.4 ± 0.0^bc^ | 0.3 ± 0.0^b^ | 0.3 | 0.8 ± 0.1^abcd^ |
| 22:5n-3 | 1.1 ± 0.1^ab^ | 1.7 ± 0.2^ab^ | 0.5 ± 0.2^b^ | 1.1 ± 0.1^ab^ | 0.9 ± 0.1^ab^ | 1.0 ± 0.1^ab^ | 1.3 ± 0.2^ab^ | 0.7 ± 0.1^ab^ | 1.1 ± 0.2^ab^ | 0.6 ± 0.1^b^ | 0.6 ± 0.1^b^ | 0.9 ± 0.1^ab^ | 0.4 ± 0.1^b^ | 0.6 ± 0.0^b^ | 0.8 ± 0.1^ab^ | 0.8 ± 0.1^ab^ | 0.6 | **2.0** **± 0.1^a^** |
| 22:5n-6 | 0.3 ± 0.0^abc^ | 0.4 ± 0.1^abc^ | 0.2 ± 0.1^bc^ | 0.2 ± 0.0^c^ | 0.2 ± 0.0^c^ | 0.3 ± 0.1^abc^ | 0.2 ± 0.0^c^ | 0.4 ± 0.1^abc^ | **0.8 ± 0.3^abc^** | 0.7 ± 0.1^abc^ | 0.7 ± 0.0^ab^ | 0.8 ± 0.1^a^ | 0.7 ± 0.1^abc^ | 0.8 ± 0.1^ab^ | 0.6 ± 0.1^abc^ | 0.6 ± 0.0^abc^ | 0.4 | 0.5 ± 0.0^abc^ |
| 22:6n-3 (DHA) | 1.2 ± 0.2^bc^ | 2.1 ± 0.3^abc^ | 1.0 ± 0.4^bc^ | 1.5 ± 0.2^abc^ | 1.0 ± 0.1^c^ | 1.4 ± 0.1^abc^ | 0.9 ± 0.2^bc^ | 1.8 ± 0.4^abc^ | 1.1 ± 0.3^abc^ | **2.8** **± 0.2^a^** | 2.0 ± 0.2^abc^ | 2.2 ± 0.2^abc^ | 2.6 ± 0.2^ab^ | 2.3 ± 0.2^abc^ | 2.2 ± 0.3^abc^ | 2.1 ± 0.1^abc^ | 1.0 | 2.4 ± 0.2^abc^ |
| Other PUFA | 1.7 ± 0.2^ab^ | 1.7 ± 0.4^ab^ | 1.4 ± 0.5^abcd^ | 0.3 ± 0.0^cd^ | **2.3 ± 0.2^a^** | 0.5 ± 0.1^abcd^ | 0.6 ± 0.1^abcd^ | 1.0 ± 0.2^abd^ | 0.4 ± 0.0^abcd^ | 0.3 ± 0.0^cd^ | 0.2 ± 0.0^c^ | 0.3 ± 0.0^bcd^ | 0.3 ± 0.1^cd^ | 0.3 ± 0.0^cd^ | 0.4 ± 0.0^abcd^ | 0.4 ± 0.0^abcd^ | 0.2 | 0.6 ± 0.0^abcd^ |
| ΣPUFA | 15.4 ± 1.1 | 19.2 ± 1.1 | 9.7 ± 2.8 | 15.6 ± 0.9 | 19.5 ± 0.9 | 14.9 ± 0.8 | 11.9 ± 1.7 | 15.7 ± 2.2 | 14.5 ± 1.5 | 19.1 ± 0.9 | 18.9 ± 1.0 | 18.6 ± 0.5 | 16.0 ± 1.3 | 18.0 ± 0.7 | 18.1 ± 1.1 | 16.6 ± 0.6 | 16.8 | 17.5 ± 0.7 |
| Σn-3 PUFA | 6.9 ± 0.5^ab^ | **8.4 ± 0.6^a^** | 3.4 ± 1.2^ab^ | 6.5 ± 0.5^ab^ | 5.6 ± 0.4^ab^ | 6.9 ± 0.5^ab^ | 5.2 ± 0.9^ab^ | 5.4 ± 0.8^ab^ | 4.1 ± 0.6^b^ | 5.7 ± 0.6^ab^ | 4.9 ± 0.6^ab^ | 6.0 ± 0.4^ab^ | 5.9 ± 0.7^ab^ | 5.7 ± 0.3^ab^ | 7.0 ± 0.6^ab^ | 7.0 ± 0.4^ab^ | 6.2 | 6.8 ± 0.4^ab^ |
| Σn-6 PUFA | 6.7 ± 0.5^b^ | 9.1 ± 0.8^abc^ | 4.9 ± 1.2^b^ | 8.7 ± 0.5^abc^ | 11.7 ± 0.6^ac^ | 7.5 ± 0.6^bc^ | 6.1 ± 0.7^abc^ | 9.3 ± 1.3^abc^ | 10 ± 0.9^abc^ | 13.1 ± 0.6^a^ | **13.8 ± 0.6^a^** | 12.3 ± 0.3^a^ | 9.8 ± 0.8^abc^ | 12.0 ± 0.5^ac^ | 10.7 ± 0.6^abc^ | 9.2 ± 0.3^abc^ | 10.3 | 10.2 ± 0.4^abc^ |
| Σn-3/Σn-6 | **1.0 ± 0.0^a^** | 0.9 ± 0.0^abc^ | 0.7 ± 0.1^abc^ | 0.7 ± 0.0^ac^ | 0.5 ± 0.0^abc^ | 0.9 ± 0.1^a^ | 0.9 ± 0.1^ac^ | 0.6 ± 0.0^abc^ | 0.4 ± 0.0^bc^ | 0.4 ± 0.0^abc^ | 0.4 ± 0.0^b^ | 0.5 ± 0.0^abc^ | 0.6 ± 0.1^abc^ | 0.5 ± 0.0^abc^ | 0.7 ± 0.0^abc^ | 0.8 ± 0.0^abc^ | 0.6 | 0.7 ± 0.0^abc^ |
| 16:1n-7/16:0 | 0.1 ± 0.0^ab^ | 0.0 ± 0.0^abc^ | **0.2 ± 0.0^a^** | 0.0 ± 0.0^abc^ | 0.0 ± 0.0^abc^ | 0.1 ± 0.0^abc^ | 0.1 ± 0.0^a^ | 0.0 ± 0.0^bc^ | 0.0 ± 0.0^abc^ | 0.0 ± 0.0^c^ | 0.0 ± 0.0^c^ | 0.0 ± 0.0^abc^ | 0.0 ± 0.1^abc^ | 0.0 ± 0.0^c^ | 0.0 ± 0.0^abc^ | 0.1 ± 0.0^abc^ | 0.0 | 0.0 ± 0.0^c^ |
| Σ18:0 | 22.8 ± 0.5^c^ | 25.7 ± 1.4^abcd^ | 23.7 ± 1.0^cd^ | 26.7 ± 0.6^abcd^ | 26.3 ± 0.5^bcd^ | 25.3 ± 0.4^bcd^ | 24.3 ± 0.4^cd^ | 27.2 ± 2.0^abcd^ | 26.8 ± 0.3^abcd^ | **34.5 ± 0.9^a^** | 33.1 ± 0.8^a^ | 29.2 ± 0.5^abcd^ | 31.8 ± 0.3^ab^ | 29.6 ± 0.4^abd^ | 30.2 ± 0.6^abd^ | 28.7 ± 0.6^abcd^ | 30.7 | 24.2 ± 0.4^bcd^ |
| Σ16:0/Σ18:0 | 1.8 ± 0.1^a^ | 1.7 ± 0.1^ab^ | 1.8 ± 0.1^a^ | 1.8 ± 0.1^a^ | 1.6 ± 0.1^ab^ | 1.8 ± 0.1^a^ | 1.6 ± 0.1^ab^ | 1.8 ± 0.4^ab^ | 1.4 ± 0.1^ab^ | 1.2 ± 0.1^b^ | 1.2 ± 0.1^ab^ | 1.6 ± 0.1^ab^ | 1.4 ± 0.0^ab^ | 1.6 ± 0.1^ab^ | 1.5 ± 0.1^ab^ | 1.7 ± 0.1^ab^ | 1.5 | **2.0 ± 0.2^a^** |
| DHA/EPA | 0.4 ± 0.0^de^ | 0.5 ± 0.0^abcde^ | 0.8 ± 0.1^abcde^ | 0.5 ± 0.0^bde^ | 0.3 ± 0.0^d^ | 0.4 ± 0.1^de^ | 0.5 ± 0.0^bde^ | 1.0 ± 0.1^abce^ | 1.1 ± 0.0^abcde^ | **2.1 ± 0.5^ac^** | 1.3 ± 0.1^ac^ | 1.2 ± 0.1^abc^ | 1.4 ± 0.3^abce^ | 1.0 ± 0.1^abce^ | 0.8 ± 0.1^abcde^ | 0.7 ± 0.0^abcde^ | 0.3 | 1.3 ± 0.1^c^ |
| BrFA | 0.6 ± 0.0^ab^ | 0.3 ± 0.0^abcd^ | 1.0 ± 0.2^a^ | 0.1 ± 0.0^bcd^ | 0.1 ± 0.0^d^ | 0.2 ± 0.0^abcd^ | 0.3 ± 0.0^abc^ | 0.1 ± 0.0^bcd^ | **2.2 ± 0.5^a^** | 0.2 ± 0.0^abcd^ | 0.2 ± 0.0^abcd^ | 0.2 ± 0.0^abcd^ | 0.5 ± 0.1^ab^ | 0.2 ± 0.0^abcd^ | 0.2 ± 0.0^abcd^ | 0.2 ± 0.0^abcd^ | 0.1 | 0.1 ± 0.0^cd^ |
| 18:1n-7/18:1n-9 | 0.3 ± 0.0^abcd^ | 0.3 ± 0.0^abcd^ | 0.7 ± 0.1^ab^ | 0.3 ± 0.0^bcd^ | 0.2 ± 0.0^cd^ | 0.3 ± 0.0^abcd^ | 0.4 ± 0.1^abcd^ | 0.1 ± 0.0^d^ | **0.8 ± 0.1^a^** | 0.5 ± 0.0^abc^ | 0.7 ± 0.0^a^ | 0.6 ± 0.0^ab^ | 0.7 ± 0.1^ab^ | 0.5 ± 0.0^abcd^ | 0.6 ± 0.0^abc^ | 0.4 ± 0.0^abcd^ | 0.5 | 0.3 ± 0.0^bcd^ |
