# Supplementary material for: Characterizing the trophic ecology of herbivorous coral reef fishes using stable isotope and fatty acid biomarkers
Source: PLoS One. 2025 Jun 30;20(6):e0327594. doi: 10.1371/journal.pone.0327594 (PMC12208496; doi:10.1371/journal.pone.0327594)
Supplement: S5 Table — The results are shown as the median (50% quartile) and the associated 90% Bayesian credible intervals (BCI) of diet proportions in brackets. Endoliths pooled endoliths and dense turf + endoliths and sparse turf sources. Var.: total variation explained by the model. Highest median contributions for each taxon are shown in bold type. These data are depicted visually in Fig 6a. Note that consumer data lying outside the mixing source polygon (see Fig 3b) were excluded from these models. (DOCX) [file pone.0327594.s011.docx]

| **Stable isotope-based mixing models** |  |  | |  | |  | |  |
| --- | --- | --- | --- | --- | --- | --- | --- | --- |
|  |  | **Dense turf** | | **Macroalgae** | | **Endoliths** | |  |
|  | **Sample (muscle tissue)** | **Median** | **90% BCI** | **Median** | **90% BCI** | **Median** | **90% BCI** | **Var.** |
| Acanthuridae | *Acanthurus lineatus* | **0.439** | (0.338 – 0.535) | 0.428 | (0.342 – 0.528) | 0.124 | (0.050 – 0.236) | 99.1% |
|  | *A. nigrofuscus* | 0.342 | (0.164 – 0.498) | **0.522** | (0.385 – 0.698) | 0.112 | (0.016 – 0.326) | 97.6% |
|  | *Ctenochaetus striatus* | **0.519** | (0.345 – 0.718) | 0.183 | (0.092 – 0.283) | 0.294 | (0.084 – 0.490) | 99.6% |
|  | *Naso tonganus* | 0.191 | (0.070 – 0.324) | **0.669** | (0.531 – 0.817) | 0.118 | (0.015 – 0.313) | 97.8% |
|  | *N. unicornis* | **0.933** | (0.845 – 0.978) | 0.037 | (0.011 – 0.093) | 0.025 | (0.003 – 0.096) | 99.5% |
|  | *Zebrasoma velifer* | 0.266 | (0.118 – 0.387) | **0.694** | (0.575 – 0.844) | 0.031 | (0.006 – 0.101) | 99.1% |
| Kyphosidae | *Kyphosus cinerascens* | 0.264 | (0.077 – 0.507) | **0.422** | (0.244 – 0.635) | 0.285 | (0.028 – 0.611) | 97.1% |
|  | *K. vaigiensis* | 0.080 | (0.021 – 0.192) | 0.238 | (0.055 – 0.473) | **0.672** | (0.429 – 0.874) | 99.0% |
| Labridae (Scarinae) | *Chlorurus microrhinos* | **0.838** | (0.642 – 0.947) | 0.083 | (0.023 – 0.187) | 0.058 | (0.008 – 0.261) | 97.9% |
|  | *C. spilurus* | **0.860** | (0.705 – 0.952) | 0.064 | (0.018 – 0.139) | 0.062 | (0.008 – 0.224) | 98.6% |
|  | *Scarus frenatus* | **0.533** | (0.340 – 0.752) | 0.174 | (0.082 – 0.279) | 0.284 | (0.058 – 0.507) | 99.1% |
|  | *S. ghobban* | 0.067 | (0.017 – 0.167) | **0.867** | (0.728 – 0.959) | **0.048** | (0.006 – 0.181) | 98.2% |
|  | *S. niger* | **0.552** | (0.408 – 0.669) | 0.345 | (0.249 – 0.454) | 0.091 | (0.014 – 0.250) | 98.8% |
|  | *S. rivulatus* | **0.736** | (0.615 – 0.841) | 0.201 | (0.111 – 0.291) | 0.052 | (0.009 – 0.174) | 98.9% |
|  | *S. schlegeli* | **0.738** | (0.640 – 0.829) | 0.223 | (0.137 – 0.313) | 0.032 | (0.006 – 0.106) | 99.3% |
|  | *S. spinus* | **0.849** | (0.504 – 0.956) | 0.068 | (0.017 – 0.214) | 0.060 | (0.007 – 0.353) | 97.7% |
| Siganidae | *Siganus doliatus* | 0.176 | (0.045 – 0.328) | **0.771** | (0.619 – 0.924) | 0.037 | (0.006 – 0.127) | 98.4% |
