# Supplementary material for: Characterizing the trophic ecology of herbivorous coral reef fishes using stable isotope and fatty acid biomarkers
Source: PLoS One. 2025 Jun 30;20(6):e0327594. doi: 10.1371/journal.pone.0327594 (PMC12208496; doi:10.1371/journal.pone.0327594)
Supplement: S6 Table — The results are shown as the median (50% quartile) and the associated 90% Bayesian credible intervals (BCI) of diet proportions in brackets. Endoliths pooled endoliths and dense turf + endoliths and sparse turf + coral rubble containing endoliths sources. Var.: total variation explained by the model. Highest median contributions for each taxon are shown in bold type. These data are depicted visually in Fig 6b. (DOCX) [file pone.0327594.s012.docx]

| **Fatty acid-based mixing models** |  | **Dense turf** | | **Macroalgae** | | **Endoliths** | |  |
| --- | --- | --- | --- | --- | --- | --- | --- | --- |
|  | **Sample (muscle tissue)** | **Median** | **90% BCI** | **Median** | **90% BCI** | **Median** | **90% BCI** | **Var.** |
| Acanthuridae | *Acanthurus lineatus* | 0.360 | (0.133 – 0.687) | **0.481** | (0.133 – 0.757) | 0.140 | (0.009 – 0.455) | 98.1% |
|  | *A. nigrofuscus* | 0.199 | (0.010 – 0.562) | **0.582** | (0.223 – 0.904) | 0.168 | (0.010 – 0.535) | 94.9% |
|  | *Ctenochaetus striatus* | **0.639** | (0.301 – 0.903) | 0.181 | (0.013 – 0.452) | 0.162 | (0.006 – 0.465) | 98.2% |
|  | *Naso tonganus* | 0.137 | (0.006 – 0.383) | **0.754** | (0.507 – 0.957) | 0.081 | (0.002 – 0.278) | 97.2% |
|  | *N. unicornis* | 0.047 | (0.002 – 0.210) | **0.859** | (0.576 – 0.980) | 0.072 | (0.002 – 0.343) | 97.8% |
|  | *Zebrasoma velifer* | 0.174 | (0.018 – 0.439) | **0.647** | (0.346 – 0.894) | 0.151 | (0.009 – 0.428) | 97.2% |
| Kyphosidae | *Kyphosus cinerascens* | 0.119 | (0.009 – 0.410) | **0.687** | (0.319 – 0.931) | 0.156 | (0.008 – 0.479) | 96.2% |
|  | *K. vaigiensis* | 0.030 | (0.001 – 0.124) | **0.903** | (0.768 – 0.984) | 0.050 | (0.003 – 0.188) | 98.3% |
| Pomacanthidae | *Pomacanthus sexstriatus* | 0.089 | (0.002 – 0.448) | 0.181 | (0.010 – 0.561) | **0.681** | (0.303 – 0.941) | 95.1% |
| Labridae (Scarinae) | *Chlorurus microrhinos* | 0.078 | (0.002 – 0.301) | **0.583** | (0.161 – 0.906) | 0.322 | (0.026 – 0.746) | 98.3% |
|  | *C. spilurus* | 0.105 | (0.007 – 0.405) | **0.521** | (0.163 – 0.861) | 0.338 | (0.034 – 0.742) | 96.4% |
|  | *Scarus frenatus* | 0.144 | (0.007 – 0.458) | **0.509** | (0.151 – 0.863) | 0.319 | (0.029 – 0.693) | 97.2% |
|  | *S. ghobban* | 0.126 | (0.007 – 0.442) | 0.316 | (0.016 – 0.773) | **0.520** | (0.054 – 0.897) | 96.2% |
|  | *S. niger* | 0.103 | (0.004 – 0.381) | **0.599** | (0.106 – 0.919) | 0.271 | (0.016 – 0.768) | 97.3% |
|  | *S. rivulatus* | 0.105 | (0.005 – 0.390) | **0.466** | (0.078 – 0.841) | 0.398 | (0.066 – 0.743) | 96.9% |
|  | *S. schlegeli* | 0.071 | (0.004 – 0.311) | **0.626** | (0.206 – 0.921) | 0.275 | (0.018 – 0.701) | 97.2% |
|  | *S. spinus* | **0.985** | (0.921 – 0.997) | 0.006 | (0.000 – 0.004) | 0.007 | (0.000 – 0.051) | 99.8% |
| Siganidae | *Siganus doliatus* | 0.061 | (0.000 – 0.297) | **0.717** | (0.336 – 0.951) | 0.195 | (0.010 – 0.575) | 97.3% |
